# Structure and mechanism of the broad spectrum CRISPR-associated ring nuclease Crn4

**DOI:** 10.1038/s41467-025-67607-6

**Published:** 2025-12-15

**Authors:** Haotian Chi, Ville Hoikkala, Stephen McMahon, Shirley Graham, Tracey Gloster, Malcolm F. White

**Affiliations:** School of Biology, University of St Andrews, BMS building, North Haugh, St Andrews, Fife UK

**Keywords:** Hydrolases, X-ray crystallography, CRISPR-Cas systems

## Abstract

Type III CRISPR systems detect the presence of RNA from mobile genetic elements (MGE) in prokaryotes, providing antiviral immunity. On activation, the catalytic Cas10 subunit conjugates ATP to form cyclic oligoadenylate (cOA) signalling molecules that activate ancillary effectors, providing an immune response. Cellular ring nucleases degrade cOA to reset the system. Here, we describe the structure and mechanism of a new family of ring nucleases, Crn4, associated with type III-D CRISPR systems. The crystal structure of Crn4 reveals a small homodimeric protein with a fold unrelated to any known ring nuclease or, indeed, any known protein structure. Crn4 degrades a wide range of cOA species to linear oligoadenylates in vitro and ameliorates type III CRISPR immunity in vivo. Phage and plasmids also encode Crn4 orthologues that may function as anti-CRISPRs. These observations expand our understanding of ring nucleases and reveal a new protein fold for cyclic nucleotide recognition.

## Abstract (web summary)

Ring nucleases are enzymes that degrade cyclic nucleotide signalling molecules generated by prokaryotic antiviral systems. Chi et al. report the structure and mechanism of a new class of ring nuclease, Crn4, with a very broad substrate specificity.

## Introduction

CRISPR-Cas systems provide adaptive immunity against invading mobile genetic elements (MGE) in prokaryotes^1^. Type III CRISPR-Cas systems comprise a ribonucleoprotein effector complex programmed with CRISPR RNA (crRNA) to detect invading RNA from viruses and other MGE^2^. Target RNA binding activates the catalytic Cas10 subunit, which in approximately 90% of cases harbours a specialised polymerase domain for the synthesis of cyclic oligoadenylate (cOA) molecules^3–6^. cOA acts as a second messenger, signalling viral infection in the cell by binding and activating a wide range of ancillary defence proteins (reviewed in ref. ^7^). Cleavage of bound target RNA deactivates the Cas10 cyclase activity^8^ and extant cOA molecules are degraded by enzymes known as ring nucleases^9^.

The Csm6/Csx1 family of type III CRISPR ancillary effectors use a dimeric CARF domain for cOA binding, resulting in allosteric activation of their HEPN-family ribonuclease domains^5,6^. Some of these effectors also degrade cA_4_ or cA_6_ in their CARF domains, providing a mechanism for auto-deactivation of the CRISPR immune response^10–14^. The effectors Cami1 and CalpL, which detect cA_4_ via CARF and SAVED domains, respectively, also possess intrinsic ring nuclease activity^15–17^.

The first extrinsic ring nuclease to be described, Crn1, was purified from *Saccharolobus solfataricus*, revealing a small protein with a dimeric CARF domain for cOA recognition. Crn1 slowly degrades cyclic tetra-adenylate (cA_4_) to linear di-adenylate products^18^. A second family of extrinsic ring nucleases, Crn2, use a dimeric domain unrelated to CARF proteins to bind and degrade cA_4_. Crn2 is found both in CRISPR defence operons^19–21^ and in viral genomes, where it functions as an anti-CRISPR (Acr) by rapidly degrading cA_4_ to neutralise cellular defences^21^. A third ring nuclease family, Crn3, uses a fold related to CARF domains to sandwich cA_4_ in protein tetramers, degrading the signalling molecule in a metal-dependent reaction to linear di-adenylates^22,23^. Finally, a group of related proteins (Csx15, Csx16 and Csx20) were predicted to act as ring nucleases^24^. Recently, we demonstrated that Csx16 and Csx20 are cA_4_-specific ring nucleases, and showed that the majority of cA_4_- and cA_6_-signalling type III CRISPR systems include a means to degrade their activator^25^. This has important implications for cellular outcomes on viral infection, as it provides a means to avoid programmed cell death or growth arrest^25,26^.

We previously reported a systematic bioinformatic analysis of type III CRISPR systems, which resulted in the identification of a new effector protein, Csm6-2, which is activated by cyclic hexa-adenylate (cA_6_) and cleaves RNA non-specifically^3^. Csm6-2 is typically found associated with a CorA-family trans-membrane effector, and in some instances, the two proteins are fused. The CRISPR-associated CorA effector from *Bacteroides fragilis* was previously shown to be activated by SAM-AMP—a signalling molecule derived from conjugation of ATP and S-adenosyl methionine^27^, but systems encoding both Csm6-2 and CorA may signal via cA_6_^3^.

Here, we show that these CRISPR loci include a small open reading frame encoding a hypothetical protein, hereafter named Crn4 (CRISPR ring nuclease 4), which degrades cA_3_, cA_4_ and cA_6_ to a range of linear products. The crystal structure of Crn4 reveals a novel, dimeric fold with conserved histidine and arginine residues that are essential for catalysis. Crn4 can partially relieve CRISPR-mediated immunity in a plasmid challenge assay, consistent with a function as a cellular ring nuclease, and is encoded in some phage and plasmid genomes where it likely functions as an Acr.

## Results

### Identification of the CRISPR associated protein Crn4

**Fig. 1:**
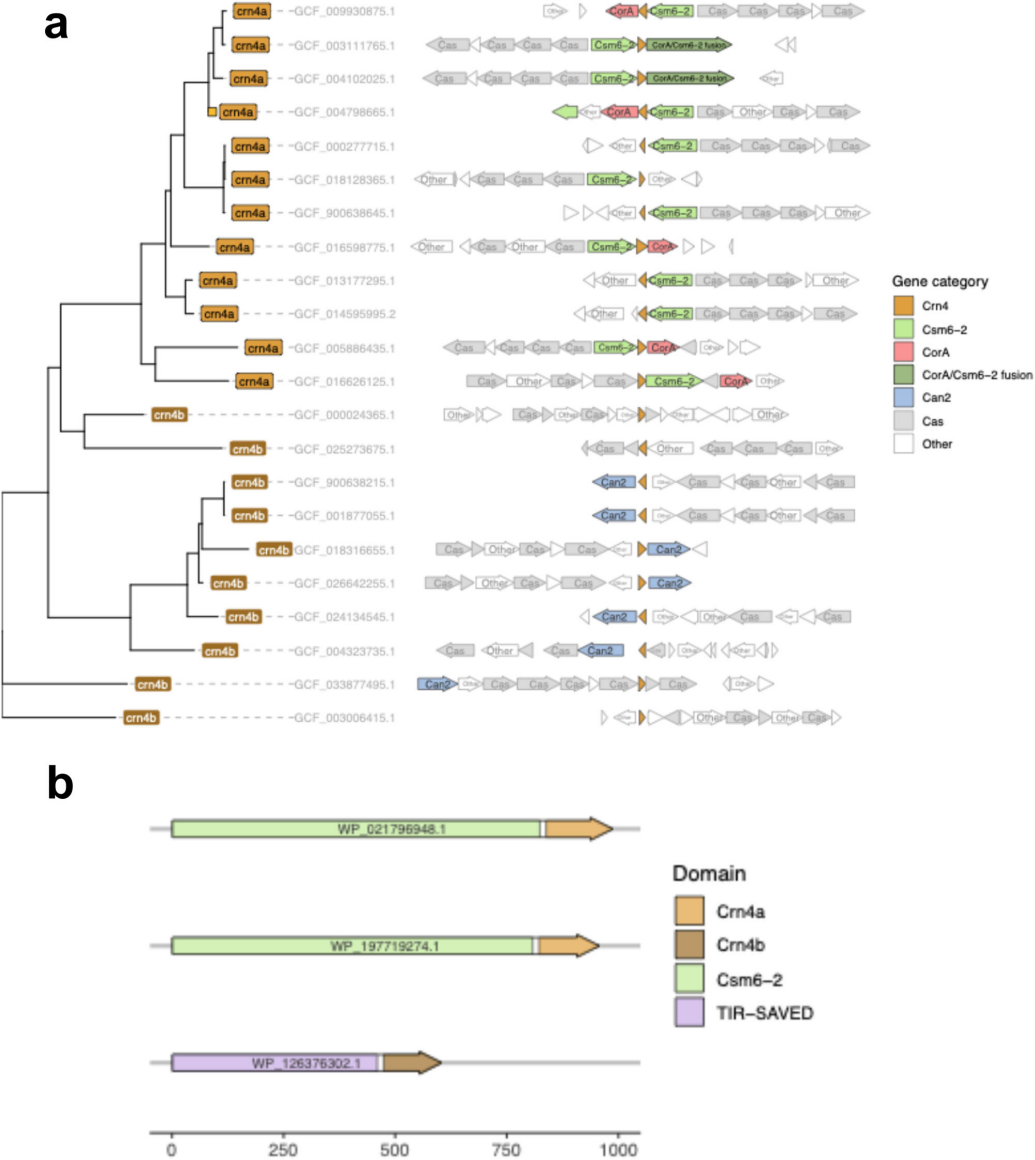
Bioinformatic analysis of Crn4. **a** A phylogenetic tree of Crn4 homologues found in type III CRISPR-Cas loci of complete prokaryotic genomes in NCBI. The subfamily of each Crn4, (**a** or **b**) is labelled on the end nodes followed by NCBI genomic accession numbers. The closest homologue (94% similarity) of the Crn4a tested experimentally in this study is marked by a small square. The genomic neighbourhoods of each Crn4, defined by CRISPR-Cas locus boundaries, is shown on the right, with key effectors highlighted as shown in the legend. **b** Three examples of Crn4 fused to the C-terminus of type III CRISPR-Cas effector proteins Csm6-2 and TIR-SAVED. Protein accession numbers are shown on the annotations.

Csm6-2, a fused Csm6 orthologue that is activated by cA_6_ and cleaves RNA, was recently identified from a bioinformatic analysis of type III CRISPR loci^3^. A systematic scan of the regions neighbouring Csm6-2 revealed a small open reading frame (ORF) encoding a hypothetical protein of unknown function in 12 CRISPR type III-D loci. In 5 of the 12 instances, a CorA membrane effector was also present (Fig. 1a). In two cases, the ORF was fused with Csm6-2, and in one instance it was fused with the effector TIR-SAVED (Fig. 1b)^3,28^. A recent bioinformatic study identified this ORF, named Unk01, as a potential ring nuclease^24^.

Using hidden Markov model (HMM) profiles based on these 12 homologues, we identified 10 additional homologues in our dataset, which were associated with the cA_4_-activated effector Can2, and two homologues with no recognisable effector in the CRISPR locus. Phylogenetic analysis divided the 22 homologues into two distinct groups: group “a” associated with Csm6-2/CorA, and group “b” associated with Can2^29,30^ or lacking a recognizable effector (Fig. 1a). The small size of the predicted proteins, combined with their consistent association with cA_4_ and cA_6_ activated effectors (either through adjacency or fusion) and prevalence on MGEs supported the prediction that Unk01 was a ring nuclease. Following established nomenclature, we provisionally designated this family of proteins CRISPR ring nuclease 4 (Crn4). The family was sub-divided into Crn4a and Crn4b, distinguishable as Crn4b members are smaller, lacking a ~ 10 amino acid region present in Crn4a (Supplementary Fig. 1). Crn4b is also observed in the genomes of phages and plasmids. Of the 28,137 phage genomes in the Millard database^31^, 11 contained a Crn4 homologue, while this was true for 10 out of 59,895 plasmid sequences in the database PLSDB^32^. Some of the phage and plasmid orthologues have an 60–80 amino acid N-terminal extension of unknown function (Supplementary Fig. 2). All members of the family have conserved residues including T13, H15 and R112 (*A. procaprae* Crn4a numbering), which are implicated in cOA binding and catalysis (further details below).

### Crn4a is a ring nuclease with broad specificity

**Fig. 2:**
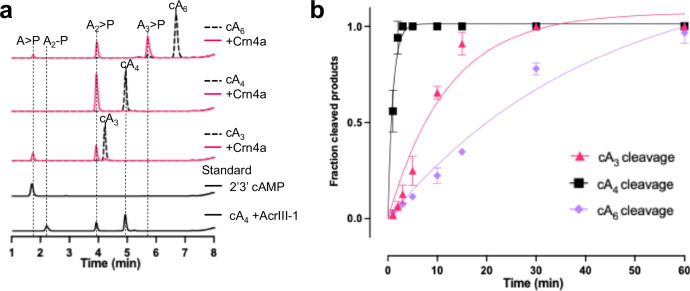
Cleavage of cOA species by Crn4a. **a** HPLC analysis of cleavage products of cA_3_, cA_4_ and cA_6_ incubated with *A. procaprae* Crn4a. 2′,3′-cAMP and a control reaction cleaving cA_4_ with AcrIII-1 to generate A_2_ > P are shown as standards. **b** Kinetic analysis of ring nuclease activity of Crn4a against cA_3_, cA_4_ and cA_6_. Following HPLC, substrate and product peaks were quantified and data plotted as fraction cleaved against time. Data points represent the means of triplicate experiments and standard deviation is shown. Source data are provided as a Source Data file.

We chose candidate Crn4a protein WP_136192672, found in the *Actinomyces procaprae* type III CRISPR locus adjacent to the previously characterised Csm6-2 protein^3^, for further study. To explore its function, we constructed a synthetic gene encoding the protein for expression in *Escherichia coli* and purified it by immobilised metal affinity and size exclusion chromatography (Supplementary Fig. 3). Crn4a was tested for ring nuclease activity by incubating the purified protein with synthetic cOA species under multiple turnover (substrate excess) conditions in EDTA, followed by HPLC analysis (Fig. 2a). Crn4a cleaved all three cOA species tested, cA_6_, cA_4_ and cA_3_, generating linear products. cA_6_ was degraded with a rate constant of 0.024 ± 0.007 min^−1^ (Fig. 2b and Supplementary Fig. 4), cA_3_ slightly faster (0.083 ± 0.02 min^−1^) and cA_4_ was degraded much more rapidly (0.98 ± 0.1 min^−1^). cA_4_ and cA_6_ were cleaved on both sides of the ring, yielding linear 2′,3′-cyclic phosphate cA_2_ > P and cA_3_ > P products, respectively (Fig. 2a and Supplementary Fig. 4). This suggests concerted or sequential cleavage of cOA on opposite sides of the molecules, reminiscent of the viral ring nuclease AcrIII-1^21^. cA_3_, which unlike cA_4_ and cA_6_ lacks an axis of twofold symmetry, was degraded to A_2_ > P and A_1_ > P, consistent with asymmetric cleavage (Fig. 2a and Supplementary Fig. 4). The ability to cleave multiple cOA species was not observed for Crn1-3^18,21,22^. Thus, in contrast to all other CRISPR associated ring nucleases tested to date, Crn4a has broad specificity for cOA species and is the first confirmed CRISPR-associated cA_3_ ring nuclease.

**Fig. 3:**
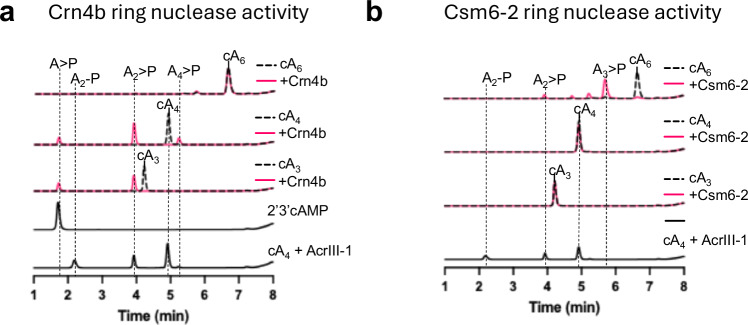
Ring nuclease activity of Crn4b and Csm6-2. **a** HPLC analysis of cleavage products of cA_3_, cA_4_ and cA_6_ incubated with Crn4b. Controls are as for Fig. 2a. **b** *A. procaprae* Csm6-2 possesses ring nuclease activity against cA_6_, but not cA_3_ or cA_4_. Source data are provided as a Source Data file.

We also cloned and expressed the gene encoding a representative of the Crn4b family (MBK8772583 from *Hyphomicrobiales bacterium*) (Supplementary Figs. 1 and 2). Crn4b degraded cA_4_ and cA_3_ but was inactive against cA_6_ (Fig. 3a). This appears consistent with the association of Crn4b with the Can2 effector, which is activated by cA_4_^29,30,33^. Although Crn4b produces the same final cA_4_ cleavage products as Crn4a, intermediate linear products (A_3_ > P and A_4_ > P) were observed at early reaction time points with cA_3_ and cA_4_, respectively (Fig. 3a and Supplementary Fig. 5), consistent with a sequential cleavage mechanism.

The close association of Crn4a with the Csm6-2 effector prompted us to test the ring nuclease activity of Csm6-2, as all Csm6 proteins studied to date possess intrinsic cA_6_ ring nuclease activities in the CARF domain of the protein^10,11,13^. Assays revealed cA_6_ degradation activity for Csm6-2 (Fig. 3b). The rate of cleavage of cA_6_, 0.026 ± 0.01 min^−1^ (Supplementary Fig. 6), was equivalent to that observed for Crn4a. However, neither cA_3_ nor cA_4_ were degraded by Csm6-2. This activity could reside in the CARF domain, HEPN domain or a combination of the two.

Given the broad specificity of Crn4a, we also tested its ability to degrade noncanonical substrates (Supplementary Fig. 7). Perhaps unsurprisingly, cA_5_, which is not known to be an activator of any CRISPR effector, was degraded efficiently by Crn4a. The CBASS signalling molecule cAAG was not a substrate, suggesting a strict specificity for adenine bases, and a high concentration of Crn4 cleaved a 12 nt polyA RNA substrate three nucleotides from the 3′ end. This suggests RNA can fold into the active site and be cleaved by the enzyme. Previously, it was demonstrated that the CARF domain of the Csx1 ribonuclease can bind linear tetra-adenylate RNA tails, which activated the HEPN domain^34^. The physiological relevance of such observations is questionable, given the slow reaction kinetics.

### Structure and mechanism of Crn4

**Fig. 4:**
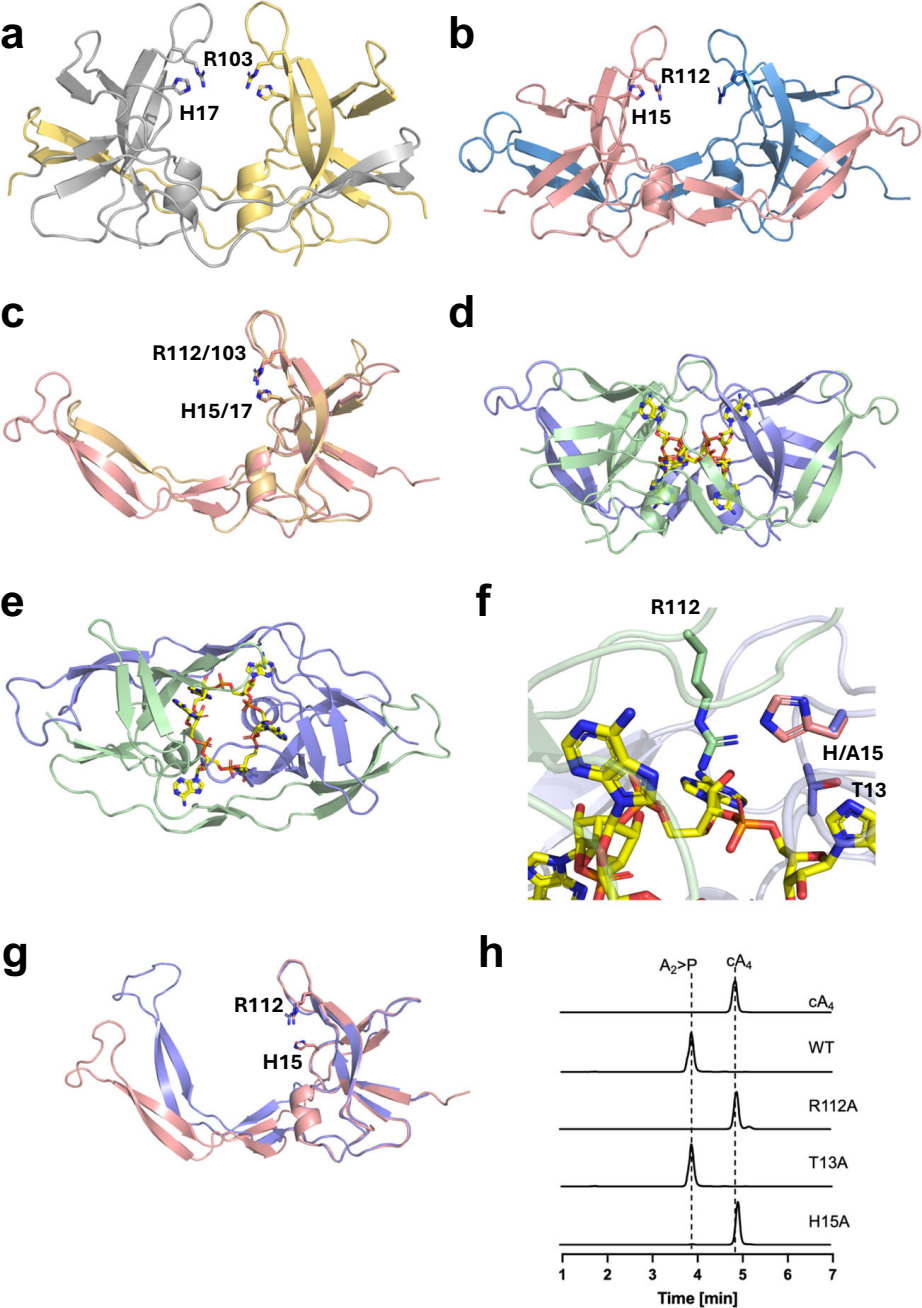
Structure and mechanism of Crn4. **a** Dimeric structure of Crn4b, with individual monomers coloured grey and gold. Each monomer has an extended β-hairpin arm wrapping around the other. The conserved residues H17 and R103 are positioned on loops above a central cavity. **b** Dimeric structure of Crn4a, with individual monomers coloured pink and blue. The conserved residues H15 and R1023 are positioned on loops above a central cavity. **c** Superimposition of a monomer of apo Crn4b (gold) and apo Crn4a (pink). Crn4a has a longer β-hairpin compared to Crn4b. The conserved residues H17 and R103 (Crnb4b) and H15 and R112 (Crn4a) are shown. **d** Dimeric structure of Crn4a in complex with cA_6_, with monomers coloured mauve and green. cA_6_ bound to Crn4a is shown in stick representation, with carbons coloured yellow. **e** Top-down view of the Crn4a dimer in complex with cA_6_ (same colouring as **d**), highlighting how the extended β-hairpin wraps around the neighbouring monomer. **f** Active site of Crn4a in complex with cA_6_, with monomers coloured green and mauve. R112 is suitably positioned to participate in cA_6_ binding and catalysis. A15 (Crn4a mutant in ligand-bound structure) is shown in mauve and H15 from the apo Crn4a structure is shown in pink; there is space for the histidine to swing into place to play a role in catalysis. T13 sits behind H15. **g** Superimposition of a monomer of apo Crn4a (pink) and Crn4a bound to cA_6_ (mauve), illustrating the movement of the β-hairpin upon binding of the ligand. Residues H15 and R112 are shown. **h** HPLC analysis of cleavage products of cA_4_ incubated with wild type Crn4a and T13A, H15A and R112A variants. Mutagenesis of H15 and R112 abolished ring nuclease activity, while the T13A variant was still active.

To characterise the structure of Crn4, we crystallised the wild-type Crn4a and Crn4b proteins in apo-form, along with the Crn4a H15A variant in the presence of cA_6_. We collected X-ray diffraction data to 2.34, 1.09 and 1.44 Å resolution, respectively. The structures were solved using molecular replacement with the AlphaFold predicted structure as the model. The structures reveal a new protein fold, with no hits identified by the DALI server^35^ with Z-scores higher than 2.6. The core structure comprises four (Crn4b; Fig. 4a) or five (Crn4a; Fig. 4b) pairs of beta strands, one parallel and the others anti-parallel, and one short alpha helix. Both proteins are dimeric, and strikingly, an extended β-hairpin, comprising one (Crn4b) or two (Crn4a) two-stranded anti-parallel beta sheets, from one monomer wraps around the neighbouring subunit, which is largely facilitated by hydrophobic interactions between the two molecules (Fig. 4a, b). The structures of apo Crn4a and Crn4b superimpose with an RSMD of 2.7 Å over 128 Cα residues. The only significant difference is the length of the hairpin loop, which is around 10 residues longer (at the N-terminus of loop) in Crn4a. The loop is unstructured in both proteins, and Crn4a and Crn4b have low sequence conservation in this region (Fig. 4c and Supplementary Fig. 8).

Upon structure solution, there was clear electron density visible in the maximum likelihood/σA weighted *F*_obs_-*F*_calc_ electron density map at 3σ corresponding to a molecule of cA_6_ bound to Crn4a that originated from the crystals co-crystallised with the ligand (Supplementary Fig. 9A). The structure of Crn4a in complex with cA_6_ shows the ligand binds at the dimer interface (ie. one molecule of cA_6_ to two protein molecules) in a symmetrical compact arrangement, with two adenine bases oriented upwards, two downwards and two roughly axial to the plane of the cA_6_ ring (Fig. 4d, e and Supplementary Fig. 9B). This differs from the conformation of cA_6_ bound to *Streptococcus thermophilus* Csm6, where four of the six adenine bases were in the plane of the ring (Supplementary Fig. 9C)^36^.

In Crn4a, cA_6_ hydrogen bonds with the main chain atoms of R92, E16, P77, A70, and side chain hydroxyl of S91, and a phosphate moiety forms an electrostatic interaction with R112 (Supplementary Fig. 9D). In addition, there is pi–pi stacking between an adenine of cA_6_ and the guanidium group of R92. R92, E16, S91 and R112 are all structurally conserved in Crn4b, as are the main chain atoms of P77 and A70, but with alternative side chains. It is noteworthy that, given the size of cA_6_, there are relatively few interactions formed with Crn4a, and the majority are with main chain atoms, suggesting the side chain functionality is not particularly important. Interestingly, R92 and R112 (or equivalent) side chains are found in a similar conformation in the two apo structures, but in a different orientation in the cA_6_ complex with Crn4a, suggesting the residues are flexible and move upon ligand binding. Stabilisation of the negative charge of cA_6_ is performed by two magnesium ions located in the centre of the cA_6_. One of these interacts with two adjacent phosphate groups and two water molecules and the other with one phosphate group and three water molecules (Supplementary Fig. 9E).

The orientation of R112 in Crn4a, and its interaction with a phosphate group in cA_6_, points to this being an important residue in phosphodiester bond cleavage (Fig. 4f). Alignment of the Crn4a H15A mutant (in complex with cA_6_) and apo Crn4a suggests that His15 would swing into a key position to participate in catalysis. A conserved threonine, T13, is positioned just behind the histidine. It is noteworthy that R112 and H15/T13 are located on different monomers in the dimer.

The inline angles formed by O^2^^′^-P-O^5^^′^ are between 100 and 110° around the ring, far from the angle of ~160° that is optimal for in-line attack by the 2′-OH nucleophile^21^. This may reflect the fact that cA_6_ is not the optimal substrate for this enzyme; additionally, the H15A variant crystallised here may differ from the wild-type enzyme in this respect.

Comparison of the apo and cA_6_-bound structures of a Crn4a monomer reveals that the globular part overlaps perfectly, but the extended β-hairpin bends upwards at an “elbow” when cA_6_ is bound (Fig. 4g). In dimeric Crn4a, this movement translates to the protein encasing the cOA upon binding (Supplementary Movies 1 and 2). This conformational change means the loops of the β-hairpin come together to sit above the central cavity where cA_6_ binds (Supplementary Fig. 9F). H15 and R112 are located on these loops, and move by 6 Å and 7 Å, respectively (Supplementary Fig. 9G), thus placing them in key positions to participate in cOA binding and/or cleavage. As H15 and R112 originate from different monomers in the Crn4a dimer, the precise loop movements from both are critical to form the active site, allowing catalysis to occur. Given the observation of relatively few interactions between cA_6_ and Crn4a (Supplementary Fig. 4D), it is possible that the protein residues displayed in the “open” form of the dimer are more important for recognition and binding of cOA. Once the cOA is enclosed in the central cavity of the “closed” dimer there is less need for interactions to guide binding or specificity, and only those important for catalysis, or for correct positioning of catalytic residues, are important.

Despite our best efforts, we were unable to crystallise a ligand-bound complex with Crn4b, or indeed Crn4a in complex with cA_3_ or cA_4_. Given the considerable movement observed for Crn4a upon binding of cA_6_ and our suggestion that specificity may largely be driven by interactions with the ‘open’ form of Crn4, direct comparison of the factors influencing specificity for substrate for each protein was not possible. Given the broad substrate specificity of the enzyme, this remains an important topic for future study.

We constructed and purified variants T13A, H15A and R112A of Crn4a to test their role in catalysis. Mutation of H15 or R112 to alanine abolished cleavage of the preferred substrate, cA_4_, after incubation at 37 °C for 60 min, while the T13A variant retained some activity (Fig. 4). Qualitatively similar data were obtained for cA_3_ and cA_6_ cleavage (Supplementary Fig. 10). These data are consistent with a role for H15 as a general acid, protonating the oxyanion leaving group during phosphodiester bond cleavage, reminiscent of H47 in AcrIII-1^21^. R112 from the neighbouring monomer likely provides a key electrostatic interaction for the cOA species during binding and catalysis.

### Crn4 is functional in vivo

To explore a functional role of Crn4 in vivo, we reconstituted *A. procaprae* Crn4a into a well-developed recombinant type IIIA CRISPR system from *Mycobacterium tuberculosis* (MtbCsm), which produces a range of cOA species for effective activation of cOA-dependent effectors to provide plasmid immunity^37^. *E. coli* C43 cells expressing MtbCsm were transformed with a pRATDuet plasmid expressing an effector protein alone or along with Crn4a and carrying a tetracycline resistance gene (*tetR*). Fewer transformants were expected when the effector was activated by cOA generated by the MtbCsm system, which was programmed with a crRNA targeting a portion of *tetR* gene. As a control, we also tested MtbCsm programmed with a crRNA targeting the pUC plasmid, which is not activated to generate cOA in this experimental setup. Effectors tested in this programmed MtbCsm system were cA_4_-activated Csx1 from *Thioalkalivibrio sulfidiphilus* (TsuCsx1)^27,38^ and cA_6_-activated effectors Csm6 from *M. tuberculosis* (MtbCsm6)^37^ and *A. procaprae* Csm6-2^3^.

**Fig. 5:**
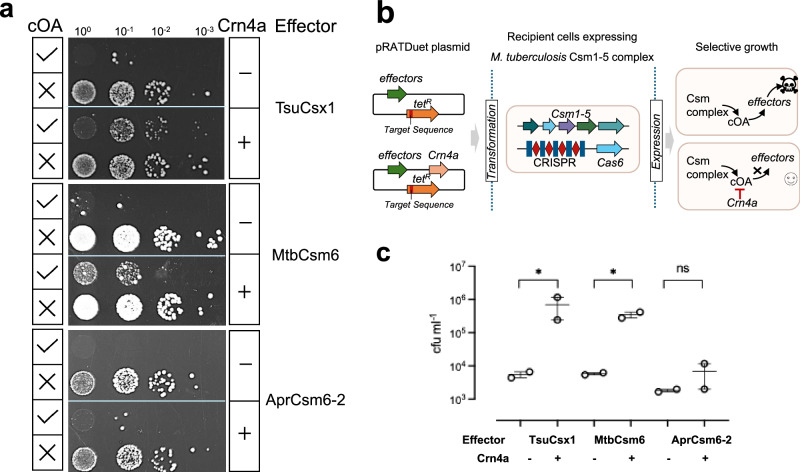
Crn4a neutralizes CRISPR immunity in a plasmid challenge assay. **a** Plasmid challenge assay. The expression of Crn4a increased cfu in cells with cA_4_-activated TsuCsx1 and cA_6_-activated MtbCsm6, consistent with degradation of those cOA species. For the Csm6-2 effector, however, no increase in cfu was observed when Crn4a was co-expressed. cOA was synthesised in the MtbCsm system programmed with crRNA targeting the *tetR* gene, while no cOA production in the MtbCsm non-target (pUC) system was set as a negative control (represented by ticks and crosses, respectively). Representative plates of two biological replicates with two technical replicates each are shown. **b** Schematic of the experimental design. **c** Colony counts for transformants in the presence or absence of Crn4a in the context of activated MtbCsm target system. Data are two biological replicates with two technical replicates each and are presented as mean ± s.d. Statistical analysis was performed using ratio paired *t* test (one-tailed *P* values listed from left to right on the graph: ^*^P = 0.0399, ^*^P = 0.0217 and ^ns^*P* = 0.25). Source data are provided as a Source Data file.

In the absence of Crn4a, all three effectors were activated on transformation of the pRATDuet plasmid, providing effective resistance to plasmid transformation, reflected in reduced colony forming units (cfu) (Fig. 5 and Supplementary Fig. 11). The presence of Crn4a effectively alleviated plasmid immunity conferred by cOA-activated TsuCsx1 and MtbCsm6 (Fig. 5), suggesting that Crn4a reduces the concentration of both cA_4_ and cA_6_ when expressed in cells. In contrast, no significant difference in colony counts was observed for the Csm6-2 effector in the presence or absence of Crn4a. This may be because Csm6-2 is itself a very active ring nuclease; alternatively, this could reflect the relative affinities of cA_6_ binding by Crn4a and Csm6-2.

## Discussion

Ring nucleases, first described in 2018^18^, are now understood to be an intrinsic aspect of type III CRISPR defence, where it seems that an ability to degrade the cOA second messengers generated by these systems is generally beneficial to the host^25^. Of the six types of specialised or stand-alone ring nucleases characterised to date, all are based on Rossmannoid domain architecture (e.g., CARF and SAVED domains)^25^. Furthermore, all of them are specific for degradation of cA_4_, the most common cOA signalling species^3^. Meanwhile, the self-limiting effectors with intrinsic ring nuclease activity also use CARF or SAVED domains to degrade either cA_6_ or cA_4_^10–17^, although the CalpL protein also has some activity against cA_3_ in vitro^16^. Here, we have described a new family of ring nucleases, Crn4, with several notable characteristics. Firstly, Crn4 displays a novel (non-Rossmann) protein fold for cOA binding. Secondly, Crn4 has ring nuclease activity against all the cOA species known to signal in type III CRISPR defence.

The structure of Crn4 appears to be unique when compared to others in the Protein DataBank, expanding our understanding of the protein folds that can bind cyclic nucleotides. The chemistry of cOA cleavage, on the other hand, is highly reminiscent of previously characterised ring nucleases, such as AcrIII-1, with both enzymes positioning an essential histidine as a likely general acid to facilitate catalysis^21^. The observation that cA_3_ is degraded efficiently by Crn4 is particularly intriguing, given that the enzyme has a dimeric organisation with two identical active sites and twofold symmetry. While this is ideal for binding of twofold symmetric substrates such as cA_4_ and cA_6_, it poses a challenge for binding and cleavage of cA_3_, which has threefold symmetry. An understanding of cA_3_ binding and cleavage at the molecular level requires a ligand-bound structure, but unfortunately, we were unsuccessful in obtaining this. In its absence, we can speculate that cA_3_ engages part of the binding site and one of the two catalytic sites, leading to asymmetric cleavage. This is probably reflected in the cleavage of polyA RNA, which is cleaved 3 nucleotides in from the 3′-terminus, suggesting partial occupancy of the binding site and considerable substrate plasticity.

The detection of Crn4b homologues in phage and plasmid genomes implies a role for these enzymes as anti-CRISPR proteins, analogous to the cA_4_-specific AcrIII-1^21^. The activity of Crn4b against both cA_3_ and cA_4_ suggests that both signalling molecules could be intercepted by these Acrs, although that remains to be confirmed. Viral nucleases targeting cA_3_ could potentially function as anti-defence enzymes against both type III CRISPR and CBASS (cyclic nucleotide based anti-phage signalling systems), each of which utilise this signalling molecule^39–42^. To date, only the broad specificity anti-defence phosphodiesterase Acb1 has been shown capable of degrading cA_3_^43^.

We first identified Crn4 as a focus for investigation based on its close association with the cA_6_-activated CRISPR effector Csm6-2^3^. As we have shown, Csm6-2 has intrinsic ring nuclease activity in its CARF domains and reaction rates in vitro are comparable to those for Crn4a, raising the question of why the latter protein is required? In fact, effectors with intrinsic ring nuclease activity are commonly found in type III CRISPR loci alongside dedicated ring nucleases^25^, suggesting there is an advantage to the host organism to have such an arrangement. One possibility is that the relatively high levels of cOA species generated by an activated type III CRISPR system^8^ necessitate a mechanism to remove these molecules when their function is no longer needed—one that does not rely on the activation, however transient, of a toxic effector protein.

In summary, these findings revealed a new class of cyclic nucleotide binding proteins, highlighting the wide distribution and diversity of ring nucleases in type III CRISPR defence.

## Methods

### Cloning

The g-blocks of the *crn4a* and *crn4b* genes were codon optimised for expression in *E. coli* and purchased from Integrated DNA Technologies. Synthetic genes were cloned into the vector pEhisV5TEV^8^ between the *Nco*I and *BamH*I sites. The *E. coli* DH5a strain was used for cloning, and sequence integrity was confirmed by sequencing (Eurofins Genomics). Site-directed mutagenesis was performed using primers with the desired mutations. All synthetic genes and primers used in this study are listed in Supplementary Table 1. For the plasmid challenge assay, the g-block of the *crn4a* gene was cloned into the MCS-1 (Multiple Cloning Site-1) of pRATDuet^37^ between the *Nde*I and *Xho*I sites, under the control of a T7 promoter. For the expression of two proteins, the synthetic gene encoding TsuCsx1^37^, MtbCsm6^37^ or Csm6-2C^3^ was cloned into the MCS-2 (Multiple Cloning Site-2) of pRATDuet-Crn4 plasmid between the *Nco*I and *Hind*III sites, under the control of pBAD promoter.

### Protein expression and purification

*E. coli* C43 (DE3) cells were transformed with plasmids for protein expression. 1 L of Luria-Broth (LB) containing cells transformed with the plasmid of interest was grown at 37 °C to an OD_600_ of 0.6–0.8. Crn4a or Crn4b expression was induced with 0.4 mM isopropyl b-D-1-thiogalactopyranoside for 4 h at 25 °C and 37 °C, respectively. Cell pellets were harvested for purification by centrifugation at 4000 × *g* (Beckman Coulter Avanti JXN-26; JLA8.1 rotor) at 4 °C for 15 min. Pellets were lysed by sonicating for 6 min with 1 min rest intervals on ice, before pelleting cell debris by ultracentrifugation at 160,000 × *g* (Beckman Coulter Optima L-90K, 70 Ti rotor) at 4 °C for 30 min. Supernatants were subsequently loaded onto a 5 mL HisTrap FF column (GE Healthcare) equilibrated with lysis buffer. After washing away unbound proteins with 20 column volumes of lysis buffer, a linear gradient elution was conducted with pump A (lysis buffer) and B (elution buffer: 50 mM Tris-HCl pH 8.0, 0.5 M NaCl, 0.5 M imidazole, and 10% glycerol). Fractions containing recombinant protein were collected and concentrated for his-tag removal by overnight dialysis with TEV protease (1 mg per 10 mg protein) in size exclusion chromatography (SEC) buffer (20 mM Tris-HCl pH 8.0, 0.25 M NaCl, 1 mM DTT, and 10% glycerol). The TEV-cleaved protein was recovered by second immobilised metal affinity chromatography (IMAC) and further purified by SEC (S200 16/60 column, GE Healthcare) in SEC buffer under isocratic flow. The identity and purity of proteins were verified using SDS-PAGE and the pure protein was aliquoted and stored at −70 °C.

### Ring nuclease activity

To examine the ring nuclease activity, enzymes were incubated with 200-fold molar excess of cOA species in 20 mM Tris-HCl, pH 7.5, 250 mM NaCl and 1 mM EDTA at 37 °C for the time indicated. For calculation of rate constants of wild type Crn4a/b and Csm6-2, 500 nM enzyme was incubated with 100 $\mu$M cA_3_, cA_4_ or cA_6_, at 37 °C for the time points 1, 2, 3, 5, 10, 15, 30 and 60 min. Reaction samples were quenched by mixing with two equivalent volumes of cold methanol and vortexed for 30 s, before centrifugation at 4 °C for 15 min. The supernatant was transferred to a new tube and vacuum dried, before resuspension in H_2_O for HPLC. Substrate and cleaved products were quantified from triplicate measurements and data were fitted using a one phase exponential model (Y = Ymax*(1-e^-k*X^)) using Prism (Version 10.2.2 (341)).

### HPLC

HPLC analysis was conducted on a UltiMate3000 UHPLC system (Thermo Fisher Scientific) equipped with an Accucore™ C18 column (Thermo Fisher Scientific 2.1 × 100 mm, particle size 2.6 ${{\rm{\mu }}}$m) for the time-course samples and a C18 column (Kinetex EVO 2.1 × 50 mm, particle size 2.6 ${{\rm{\mu }}}$m) for other analyses. The absorbance was monitored at 260 nm and the column temperature was set at 40 °C. Solvent A was 20 mM ammonium acetate, pH 8.5 and B was methanol. The flow rate was set at 0.4 ml/min for the Accucore™ C18 column and 0.3 ml/min for the EVO C18 column. Gradient elution followed the same procedure: 0–0.5 min, 1% B; 0.5–6 min, 1–15% B; 6–7 min, 100% B.

### Protein crystallisation

Crn4a H15A, at 11 mg mL^−1^, was incubated at room temperature for 30 min with 1.2 molar excess of cA_6_. Crn4a and Crn4b were used at concentrations of 13 and 15 mg mL^−1^ respectively. Immediately prior to crystallisation proteins were centrifuged at 14000 × *g*. Sitting drop vapour diffusion experiments were set up at the nanoliter scale using commercially available crystallisation screens and incubated at 293 K. Optimised Crn4a H15A with cA_6_ crystals were grown from 23.8% PEG 3350, 0.2 M magnesium chloride and 0.1 M Bis-Tris, pH 5.5. Apo Crn4a crystals were grown from 0.1 M sodium acetate, pH 4.5 and 22% v/v PEG Smear Broad (Molecular Dimensions). Crn4b crystals grew from 20% PEG 6000, 10% ethylene glycol and 0.1 M calcium chloride. Crystals were cryoprotected with 25% glycerol prior to harvesting and cryo-cooling in liquid nitrogen.

### X-ray data collection, structure solution, and refinement

X-ray data were collected at a wavelength of 0.9537 Å, 100 K, on beamline I04 at the Diamond Light Source, to 2.34 Å (apo Crn4a), 1.44 Å (Crn4a H15A with cA_6_) and 1.09 Å (apo Crn4b) resolution. Data were automatically processed using Xia2^44^. The structures were solved by phasing the data using PhaserMR^45^ in the CCP4 suite^46^, using a model generated by AlphaFold 3^47^, with initial B-factors modelled in Phenix^48^. Model refinement was achieved by iterative cycles of REFMAC5^49^, where hydrogen atoms were added for refinement but not output to the pdb files, and manual model manipulation in COOT^50^. For the Crn4a H15A with cA_6_ data, electron density for cA_6_ was clearly visible in the maximum likelihood/σA weighted *F*_obs_-*F*_calc_ electron density map at 3σ. The coordinates for cA_6_ were generated in ChemDraw (Perkin Elmer) and the library was generated using Acedrg^51^, before fitting the molecule in COOT. The quality of each structure was monitored throughout using Molprobity^52^. Data and refinement statistics are shown in Supplementary Table 2. The coordinates and raw data have been validated and deposited in the Protein Data Bank with deposition codes 9SMA for Crn4a, 9QS9 for Crn4a H15A in complex with cA_6_, and 9R7B for Crn4b.

### Plasmid challenge assay

Plasmids from the programmed type III MtbCsm system have been described, including pCsm1-5 (containing Csm interference genes *cas10*, *csm3*, *csm4* and *csm5* from *M. tuberculosis* and *csm2* from *M. canettii*)^37^, pCRISPR-TetR and pCRISPR-pUC (containing *M. tuberculosis cas6* and a CRISPR array targeting a tetracycline-resistance gene or a pUC multiple cloning site, respectively)^37^ and pRATDuet-effectors (carrying tetracycline-resistance gene and *M. tuberculosis* (Mtb) *csm6 or Thioalkalivibrio sulfidiphilus* (Tsu) *csx1*)^53^.

*E. coli* C43 (DE3) cells carrying plasmids pCsm1-5 and pCRISPR-TetR or pCRISPR-pUC were transformed with 100 ng of pRATDuet derived plasmids containing different effectors with or without a Crn4 ring nuclease. After recovering at 37  °C for 2 h, a tenfold serial dilution of cells was applied onto LB agar supplemented with 100 μg/ml ampicillin and 50 μg/ml spectinomycin for determination of cell density of recipients, onto LB agar containing additional 12.5 μg/ml tetracycline for determination of transformation efficiency and LB agar with additional 0.2% (w/v) D-lactose and 0.2 % (w/v) L-arabinose to determine the plasmid immunity mediated by cOA-dependent effectors. Plates were incubated at 37 °C overnight, before taking images of plates. Technical duplicates of two biological replicates were performed. Colonies were counted to calculate colony forming units (cfu/ml) and analysed using an unpaired *t* test to compare the difference between systems in the presence or absence of Crn4 using Prism (Version 10.2.2).

### Bioinformatic analyses

To search for Crn4 homologues in prokaryotes, we downloaded the 38,742 complete prokaryotic genomes from NCBI on March 25th 2024. For phage genomes we used the Millard database of 28,114 genomes (May 2024 release)^31^ and for plasmids the PLSDB database containing 59,895 sequences^32^. Based on our initial identification of two main clades for Crn4 homologues, separate HMM databases were created for Crn4a and Crn4b. These profiles were integrated into a previously published Snakemake pipeline developed for finding type III CRISPR-Cas loci, effectors and ring nucleases in prokaryotic and in phage genomes^3^. In short, the pipeline annotates type III CRISPR-Cas loci and searches them for ring nucleases based on HMM profiles and protein length limitations.

To create the phylogenetic tree (Fig. 1), prokaryotic Crn4a and Crn4b amino acid sequences were concatenated into one file, and aligned using Muscle^54^. A phylogenetic tree based on the alignment was created with FastTree^55^ using -wag and -gamma arguments. Two Crn4 homologues (genomes GCF_002285795.1 and GCF_002863905.1) were removed from the dataset due to missing cyclase domains in the accompanying Cas10. The tree was plotted and annotated in RStudio v. XX with ggtree^56^. The accompanying genomic neighbourhood visualisation was created by mergingh information from several annotation files output by the pipeline (CCTyper, cATyper, RN_typer) into the original NCBI general features files (.gff). The enhanced annotations were visualised using ggtree’s geom_facet wrapper for gggenes and centered around Crn4. Custom rules were used to bin the genes into the categories shown in Fig. 1. Crn4 fusions with known effector proteins were discovered by modifying the snakemake pipeline to search for Crn4 without protein length limitations.

### Reporting summary

Further information on research design is available in the Nature Portfolio Reporting Summary linked to this article.

## Supplementary information

Supplementary Information

Description of Additional Supplementary Files

Supplementary Movie 1

Supplementary Movie 2

Reporting Summary

Transparent Peer Review file

## Source data

Source Data

## Data Availability

Unless otherwise stated, all data supporting the results of this study can be found in the article, supplementary, and source data files. The protein structure coordinates and data have been deposited in the Protein Data Bank [www.pdb.org] with deposition codes 9SMA for apo Crn4a, 9QS9 for Crn4a H15A in complex with cA_6_ and 9R7B for Crn4b. Source data are provided with this paper.

## Code availability

The code used for bioinformatic analyses has been uploaded to Zenodo [10.5281/zenodo.17723392].

## References

[1] Makarova, K. S. et al. Evolutionary classification of CRISPR–Cas systems: a burst of class 2 and derived variants. *Nat. Rev. Microbiol.* **18**, 67–83 (2020). 31857715 10.1038/s41579-019-0299-x PMC8905525

[2] Stella, G. & Marraffini, L. Type III CRISPR-Cas: beyond the Cas10 effector complex. *Trends Biochem. Sci.* **49**, 28–37 (2024). 37949766 10.1016/j.tibs.2023.10.006 PMC10844953

[3] Hoikkala, V., Graham, S. & White, M. F. Bioinformatic analysis of type III CRISPR systems reveals key properties and new effector families. *Nucleic Acids Res.* **52**, 7129–7141 (2024). 38808661 10.1093/nar/gkae462 PMC11229360

[4] Wiegand, T. et al. Functional and phylogenetic diversity of Cas10 proteins. *CRISPR J.* **6**, 152–162 (2023). 36912817 10.1089/crispr.2022.0085 PMC10123807

[5] Kazlauskiene, M., Kostiuk, G., Venclovas, C., Tamulaitis, G. & Siksnys, V. A cyclic oligonucleotide signaling pathway in type III CRISPR-Cas systems. *Science* **357**, 605–609 (2017). 28663439 10.1126/science.aao0100

[6] Niewoehner, O. et al. Type III CRISPR-Cas systems produce cyclic oligoadenylate second messengers. *Nature* **548**, 543–548 (2017). 28722012 10.1038/nature23467

[7] Steens, J. A., Salazar, C. R. P. & Staals, R. H. J. The diverse arsenal of type III CRISPR-Cas-associated CARF and SAVED effectors. *Biochem. Soc. Trans.* **50**, 1353–1364 (2022). 36282000 10.1042/BST20220289 PMC9704534

[8] Rouillon, C., Athukoralage, J. S., Graham, S., Grüschow, S. & White, M. F. Investigation of the cyclic oligoadenylate signalling pathway of type III CRISPR systems. *Methods Enzymol.* **616**, 191–218 (2019). 30691643 10.1016/bs.mie.2018.10.020

[9] Athukoralage, J. S. & White, M. F. Cyclic oligoadenylate signalling and regulation by ring nucleases during type III CRISPR defence. *RNA* **27**, 855–867 (2021). 33986148 10.1261/rna.078739.121 PMC8284326

[10] Garcia-Doval, C. et al. Activation and self-inactivation mechanisms of the cyclic oligoadenylate-dependent CRISPR ribonuclease Csm6. *Nat. Commun.* **11**, 1596 (2020). 32221291 10.1038/s41467-020-15334-5 PMC7101355

[11] Athukoralage, J. S., Graham, S., Gruschow, S., Rouillon, C. & White, M. F. A type III CRISPR ancillary ribonuclease degrades its cyclic oligoadenylate activator. *J. Mol. Biol.* **431**, 2894–2899 (2019). 31071326 10.1016/j.jmb.2019.04.041 PMC6599890

[12] Jia, N., Jones, R., Yang, G., Ouerfelli, O. & Patel, D. J. CRISPR-Cas III-A Csm6 CARF domain is a ring nuclease triggering stepwise cA4 cleavage with ApA>p formation terminating RNase activity. *Mol. Cell* **75**, 944-956–e946 (2019). 31326273 10.1016/j.molcel.2019.06.014 PMC6731128

[13] Smalakyte, D. et al. Type III-A CRISPR-associated protein Csm6 degrades cyclic hexa-adenylate activator using both CARF and HEPN domains. *Nucleic Acids Res.* **48**, 9204–9217 (2020). 32766806 10.1093/nar/gkaa634 PMC7498309

[14] Du, L., Zhu, Q. & Lin, Z. Molecular mechanism of allosteric activation of the CRISPR ribonuclease Csm6 by cyclic tetra-adenylate. *EMBO J.* **43**, 304–315 (2024). 38177499 10.1038/s44318-023-00017-w PMC10897365

[15] Mogila, I. et al. Ribosomal stalk-captured CARF-RelE ribonuclease inhibits translation following CRISPR signaling. *Science* **382**, 1036–1041 (2023). 38033086 10.1126/science.adj2107

[16] Binder, S. C. et al. The SAVED domain of the type III CRISPR protease CalpL is a ring nuclease. *Nucleic Acids Res.* **52**, 10520–10532 (2024). 39166476 10.1093/nar/gkae676 PMC11417357

[17] Smalakyte, D., Ruksenaite, A., Sasnauskas, G., Tamulaitiene, G. & Tamulaitis, G. Filament formation activates protease and ring nuclease activities of CRISPR Lon-SAVED. *Mol. Cell* **84**, 4239–4255 (2024). 39362215 10.1016/j.molcel.2024.09.002

[18] Athukoralage, J. S., Rouillon, C., Graham, S., Grüschow, S. & White, M. F. Ring nucleases deactivate Type III CRISPR ribonucleases by degrading cyclic oligoadenylate. *Nature* **562**, 277–280 (2018). 30232454 10.1038/s41586-018-0557-5 PMC6219705

[19] Samolygo, A., Athukoralage, J. S., Graham, S. & White, M. F. Fuse to defuse: a self-limiting ribonuclease-ring nuclease fusion for type III CRISPR defence. *Nucleic Acids Res.* **48**, 6149–6156 (2020). 32347937 10.1093/nar/gkaa298 PMC7293037

[20] Zhang, D., Du, L., Gao, H., Yuan, C. & Lin, Z. Structural insight into the Csx1-Crn2 fusion self-limiting ribonuclease of type III CRISPR system. *Nucleic Acids Res.* **52**, 8419–8430 (2024). 38967023 10.1093/nar/gkae569 PMC11317161

[21] Athukoralage, J. S. et al. An anti-CRISPR viral ring nuclease subverts type III CRISPR immunity. *Nature* **577**, 572–575 (2020). 31942067 10.1038/s41586-019-1909-5 PMC6986909

[22] Athukoralage, J. S. et al. Tetramerisation of the CRISPR ring nuclease Crn3/Csx3 facilitates cyclic oligoadenylate cleavage. *eLife* **9**, e57627 (2020). 32597755 10.7554/eLife.57627 PMC7371418

[23] Brown, S., Gauvin, C. C., Charbonneau, A. A., Burman, N. & Lawrence, C. M. Csx3 is a cyclic oligonucleotide phosphodiesterase associated with type III CRISPR-Cas that degrades the second messenger cA(4). *J. Biol. Chem.* **295**, 14963–14972 (2020). 32826317 10.1074/jbc.RA120.014099 PMC7606696

[24] Makarova, K. S. et al. Evolutionary and functional classification of the CARF domain superfamily, key sensors in prokaryotic antivirus defense. *Nucleic Acids Res.* **48**, 8828–8847 (2020). 32735657 10.1093/nar/gkaa635 PMC7498327

[25] Hoikkala, V., Chi, H., Gruschow, S., Graham, S. & White, M. F. Diversity and abundance of ring nucleases in type III CRISPR-Cas loci. *Philos. Trans. R. Soc. Lond. B Biol. Sci.* **380**, 20240084 (2025). 40904116 10.1098/rstb.2024.0084 PMC12409355

[26] Aviram, N. et al. Cas10 relieves host growth arrest to facilitate spacer retention during type III-A CRISPR-Cas immunity. *Cell Host Microbe* **32**, 2050–2062 e2056 (2024). 39626678 10.1016/j.chom.2024.11.005 PMC11708336

[27] Chi, H. et al. Antiviral type III CRISPR signalling via conjugation of ATP and SAM. *Nature* **622**, 826–833 (2023). 37853119 10.1038/s41586-023-06620-5 PMC10600005

[28] Hogrel, G. et al. Cyclic nucleotide-induced helical structure activates a TIR immune effector. *Nature* **608**, 808–812 (2022). 35948638 10.1038/s41586-022-05070-9

[29] Zhu, W. et al. The CRISPR ancillary effector Can2 is a dual-specificity nuclease potentiating type III CRISPR defence. *Nucleic Acids Res.* **49**, 2777–2789 (2021). 33590098 10.1093/nar/gkab073 PMC7969007

[30] Rostol, J. T. et al. The Card1 nuclease provides defence during type-III CRISPR immunity. *Nature* **590**, 614–629 (2021). 10.1038/s41586-021-03206-x PMC7906951 33461211

[31] Cook, R. et al. INfrastructure for a PHAge REference database: identification of large-scale biases in the current collection of cultured phage genomes. *Phage (N. Rochelle)* **2**, 214–223 (2021). 10.1089/phage.2021.0007 PMC9041510 36159887

[32] Schmartz, G. P. et al. PLSDB: advancing a comprehensive database of bacterial plasmids. *Nucleic Acids Res.* **50**, D273–D278 (2022). 34850116 10.1093/nar/gkab1111 PMC8728149

[33] McMahon, S. A. et al. Structure and mechanism of a Type III CRISPR defence DNA nuclease activated by cyclic oligoadenylate. *Nat. Commun.* **11**, 500 (2020). 31980625 10.1038/s41467-019-14222-x PMC6981274

[34] Han, W., Pan, S., Lopez-Mendez, B., Montoya, G. & She, Q. Allosteric regulation of Csx1, a type IIIB-associated CARF domain ribonuclease by RNAs carrying a tetraadenylate tail. *Nucleic Acids Res.* **45**, 10740–10750 (2017). 28977519 10.1093/nar/gkx726 PMC5737841

[35] Holm, L. DALI and the persistence of protein shape. *Protein Sci.* **29**, 128–140 (2020). 31606894 10.1002/pro.3749 PMC6933842

[36] McQuarrie, S. et al. Activation of Csm6 ribonuclease by cyclic nucleotide binding: in an emergency, twist to open. *Nucleic Acids Res.* **51**, 10590–10605 (2023). 37747760 10.1093/nar/gkad739 PMC10702470

[37] Grüschow, S., Athukoralage, J. S., Graham, S., Hoogeboom, T. & White, M. F. Cyclic oligoadenylate signalling mediates Mycobacterium tuberculosis CRISPR defence. *Nucl. Acids Res.* **47**, 9259–9270 (2019). 31392987 10.1093/nar/gkz676 PMC6755085

[38] Molina, R. et al. Structure of Csx1-cOA4 complex reveals the basis of RNA decay in Type III-B CRISPR-Cas. *Nat. Commun.* **10**, 4302 (2019). 31541109 10.1038/s41467-019-12244-z PMC6754442

[39] Steens, J. A. et al. Type III-B CRISPR-Cas cascade of proteolytic cleavages. *Science* **383**, 512–519 (2024). 38301007 10.1126/science.adk0378 PMC11220425

[40] Grüschow, S. et al. CRISPR antiphage defence mediated by the cyclic nucleotide-binding membrane protein Csx23. *Nucl. Acids Res.* **52**, 2761–2775 (2024). 38471818 10.1093/nar/gkae167 PMC11014256

[41] Lau, R. K. et al. Structure and mechanism of a cyclic trinucleotide-activated bacterial endonuclease mediating bacteriophage immunity. *Mol. Cell* **77**, 723–733 (2020). 31932164 10.1016/j.molcel.2019.12.010 PMC7065454

[42] Grüschow, S., Adamson, C. S. & White, M. F. Specificity and sensitivity of an RNA targeting type III CRISPR complex coupled with a NucC endonuclease effector. *Nucleic Acids Res.* **49**, 13122–13134 (2021). 34871408 10.1093/nar/gkab1190 PMC8682760

[43] Hobbs, S. J. et al. Phage anti-CBASS and anti-Pycsar nucleases subvert bacterial immunity. *Nature* **605**, 522–526 (2022). 35395152 10.1038/s41586-022-04716-y PMC9117128

[44] Winter, G. xia2: an expert system for macromolecular crystallography data reduction. *J. Appl. Crystallogr.* **43**, 186–190 (2010).

[45] McCoy, A. J. et al. Phaser crystallographic software. *J. Appl. Crystallogr.* **40**, 658–674 (2007). 19461840 10.1107/S0021889807021206 PMC2483472

[46] Winn, M. D. et al. Overview of the CCP4 suite and current developments. *Acta Crystallogr. D. Biol. Crystallogr.* **67**, 235–242 (2011). 21460441 10.1107/S0907444910045749 PMC3069738

[47] Abramson, J. et al. Accurate structure prediction of biomolecular interactions with AlphaFold 3. *Nature* **630**, 493–500 (2024). 38718835 10.1038/s41586-024-07487-w PMC11168924

[48] Liebschner, D. et al. Macromolecular structure determination using X-rays, neutrons and electrons: recent developments in Phenix. *Acta Crystallogr. D. Struct. Biol.* **75**, 861–877 (2019). 31588918 10.1107/S2059798319011471 PMC6778852

[49] Murshudov, G. N. et al. REFMAC5 for the refinement of macromolecular crystal structures. *Acta Crystallogr. D.* **67**, 355–367 (2011). 21460454 10.1107/S0907444911001314 PMC3069751

[50] Casanal, A., Lohkamp, B. & Emsley, P. Current developments in Coot for macromolecular model building of electron cryo-microscopy and crystallographic data. *Protein Sci.* **29**, 1069–1078 (2020). 31730249 10.1002/pro.3791 PMC7096722

[51] Long, F. et al. AceDRG: a stereochemical description generator for ligands. *Acta Crystallogr. D. Struct. Biol.* **73**, 112–122 (2017). 28177307 10.1107/S2059798317000067 PMC5297914

[52] Chen, V. B. et al. MolProbity: all-atom structure validation for macromolecular crystallography. *Acta Crystallogr. D. Biol. Crystallogr.* **66**, 12–21 (2010). 20057044 10.1107/S0907444909042073 PMC2803126

[53] Athukoralage, J. S. et al. The dynamic interplay of host and viral enzymes in type III CRISPR-mediated cyclic nucleotide signalling. *eLife* **9**, e55852 (2020). 32338598 10.7554/eLife.55852 PMC7213978

[54] Edgar, R. C. Muscle5: high-accuracy alignment ensembles enable unbiased assessments of sequence homology and phylogeny. *Nat. Commun.* **13**, 6968 (2022). 36379955 10.1038/s41467-022-34630-w PMC9664440

[55] Price, M. N., Dehal, P. S. & Arkin, A. P. FastTree 2-approximately maximum-likelihood trees for large alignments. *PLoS ONE* **5**, e9490 (2010). 20224823 10.1371/journal.pone.0009490 PMC2835736

[56] Yu, G. Using ggtree to visualize data on tree-like structures. *Curr. Protoc. Bioinforma.* **69**, e96 (2020). 10.1002/cpbi.96 32162851

