# Supplementary material for: Structure and mechanism of the broad spectrum CRISPR-associated ring nuclease Crn4
Source: Nat Commun. 2025 Dec 15;17:889. doi: 10.1038/s41467-025-67607-6 (PMC12830947; doi:10.1038/s41467-025-67607-6)
Supplement: Supplementary file 1 — Supplementary Information [file 41467_2025_67607_MOESM1_ESM.pdf]

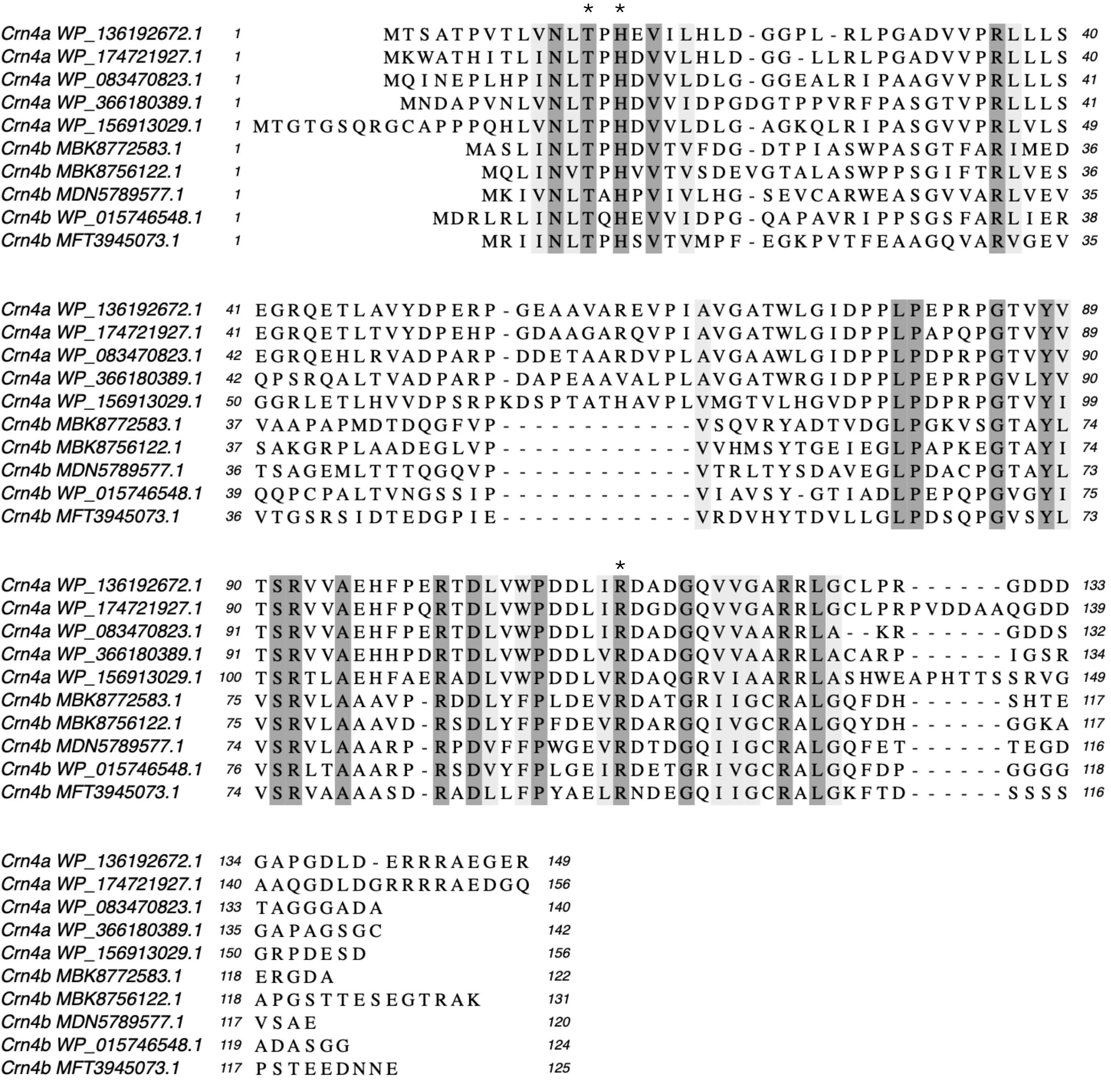

Supplementary Figure 1. Multiple sequence alignment of a selection of Crn4a and Crn4b proteins. *A. procaprae* Crn4a (WP_136192672.1) and Crn4b (MBK8772583.1) are studied biochemically in this manuscript. Conserved residues are shaded. T12, H15 and R112 (AprCrn4a numbering) are highlighted with asterisks.

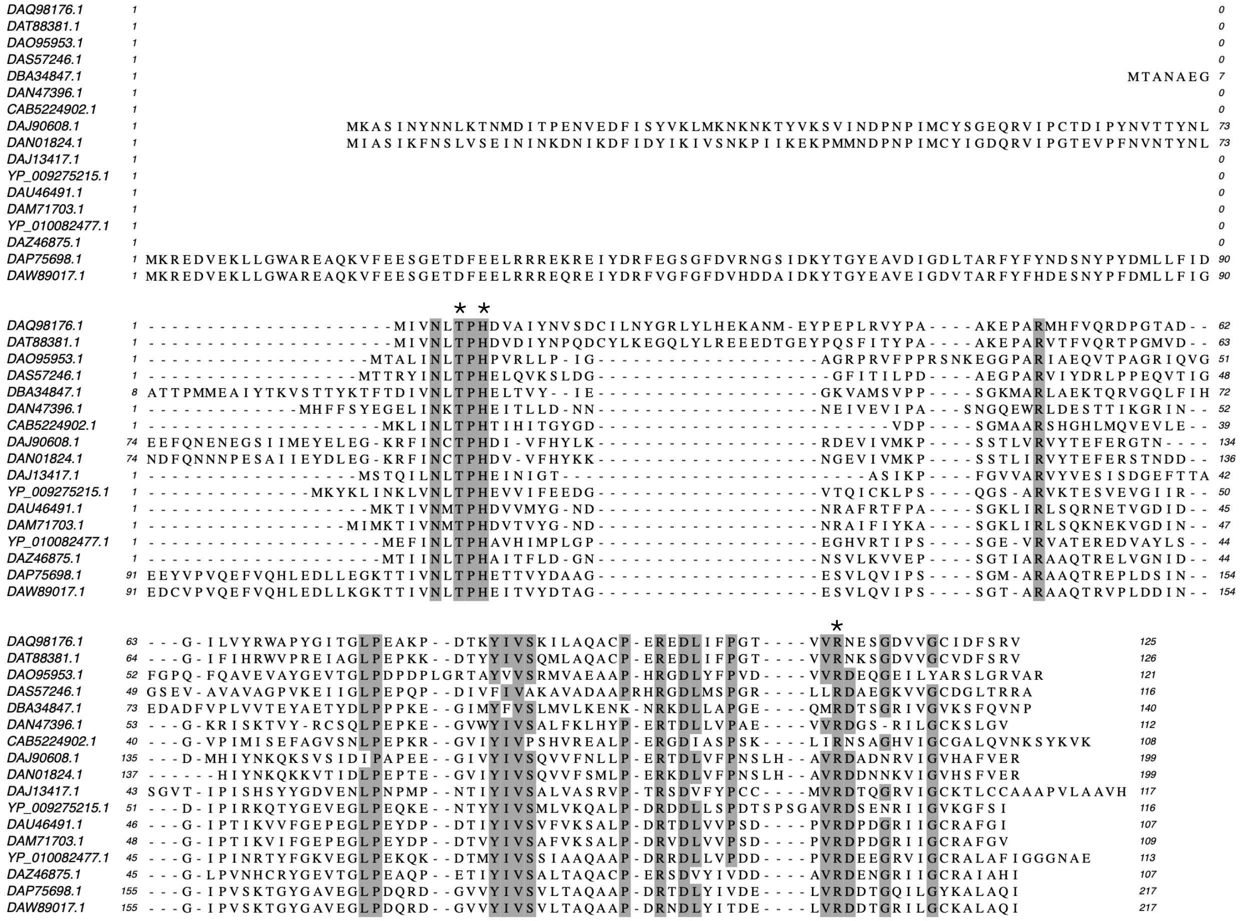

Supplementary figure 2. Sequence alignment of bacteriophage and plasmid Crn4 orthologues. T12, H15 and R112 (AprCrn4a numbering) are highlighted with asterisks. Some members of the family have an 80-100 aa N-terminal extension.

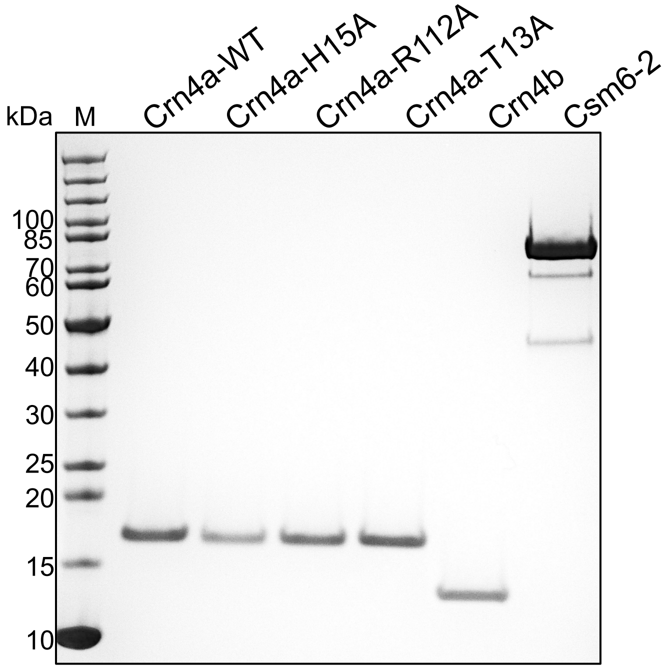

Supplementary Figure 3. SDS-PAGE analysis of purified Crn4a, Crn4b and Csm6-2 proteins. The observed mass of Crn4a, Crn4b, and Csm6-2 on the gel is about 16, 14, and 86 kDa respectively, consistent with their theoretical mass. M is the molecular weight marker with sizes indicated.

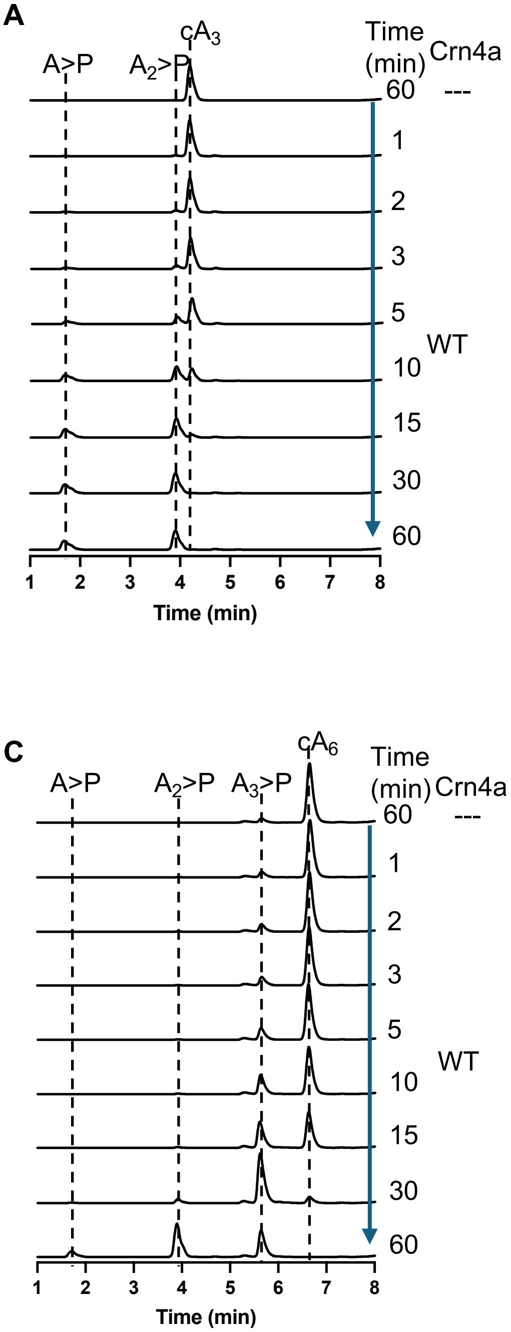

Supplementary Figure 4. Time course of Crn4a ring nuclease activity.
A. HPLC analysis of cA_3-cleavage activity of Crn4a wild-type. 0.5 μM Crn4a is incubated with 100 μM cA_3, cA_4 in **B** and cA_6 in **C** at 37 °C for the time points 1, 2, 3, 5, 10, 15, 30 and 60 min. Representative HPLC traces of three replicates are present. Source data are provided as a Source Data file.

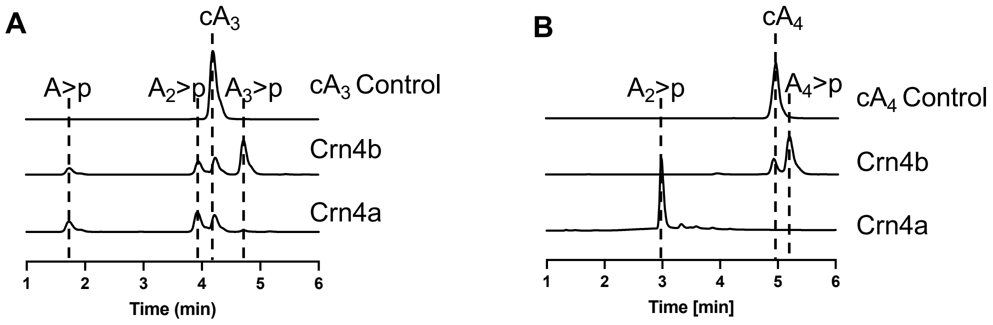

Supplementary Figure 5. Comparison of ring nuclease activity of Crn4a and Crn4b.
A. HPLC analysis of cA_3-cleavage activity of Crn4a and Crn4b wild-type. 0.5 μM Crn4 is incubated with 100 μM cA_3 (cA_4 in **B)** at 37 °C for 10 min. Source data are provided as a Source Data file.

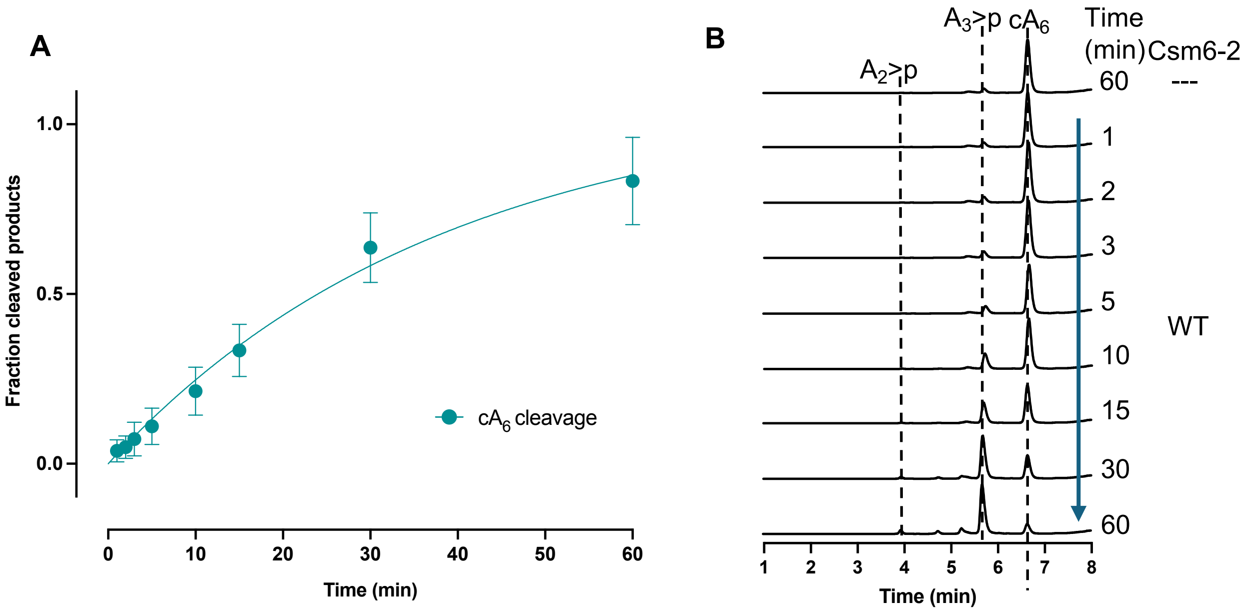

Supplementary Figure 6. Csm6-2 ring nuclease activity against cA_6. A. Kinetic analysis of cA_6 cleavage activity. HPLC peaks of substrate and products were quantified and data plotted as fraction cleaved against time. Data points are shown as the means of triplicate experiments and standard deviation is shown. **B.** Representative HPLC traces of triplicate time course experiments are present. Source data are provided as a Source Data file.

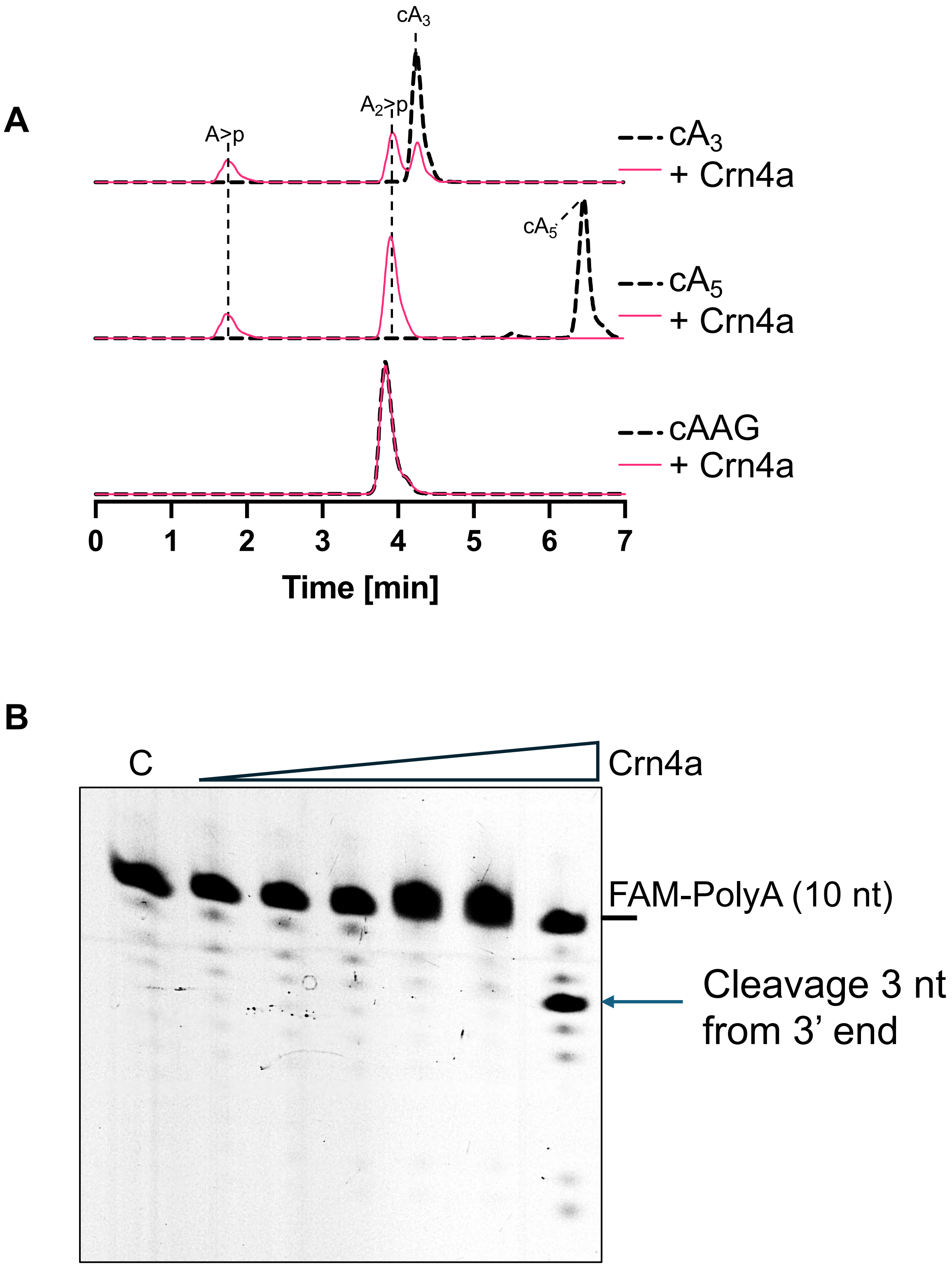

Supplementary Figure 7. Cleavage of non-canonical substrates by Crn4a.
A. HPLC analysis of cyclic nucleotides cleavage activity. Crn4a (0.5 µM) was incubated with cA_3 (100 µM), cA_5 (10 µM) or cAAG (100 µM, 3'-5' phosphodiester linkage), at 37°C for 15 min. No cAAG cleavage activity was observed, but cA_5 was degraded efficiently **B.** Linear PolyA cleavage activity. A 5'-end FAM labelled PolyA of 10 nucleotides was incubated with Crn4a (20, 50, 100, 250, 500 and 5000 nM) at 37°C for 60 min. Control sample (C) shows incubation in the absence of enzyme. PolyA cleavage activity (positioned 3 nt from the 3' end of the RNA) was only observed at the high concentration of Crn4a. Source data are provided as a Source Data file.

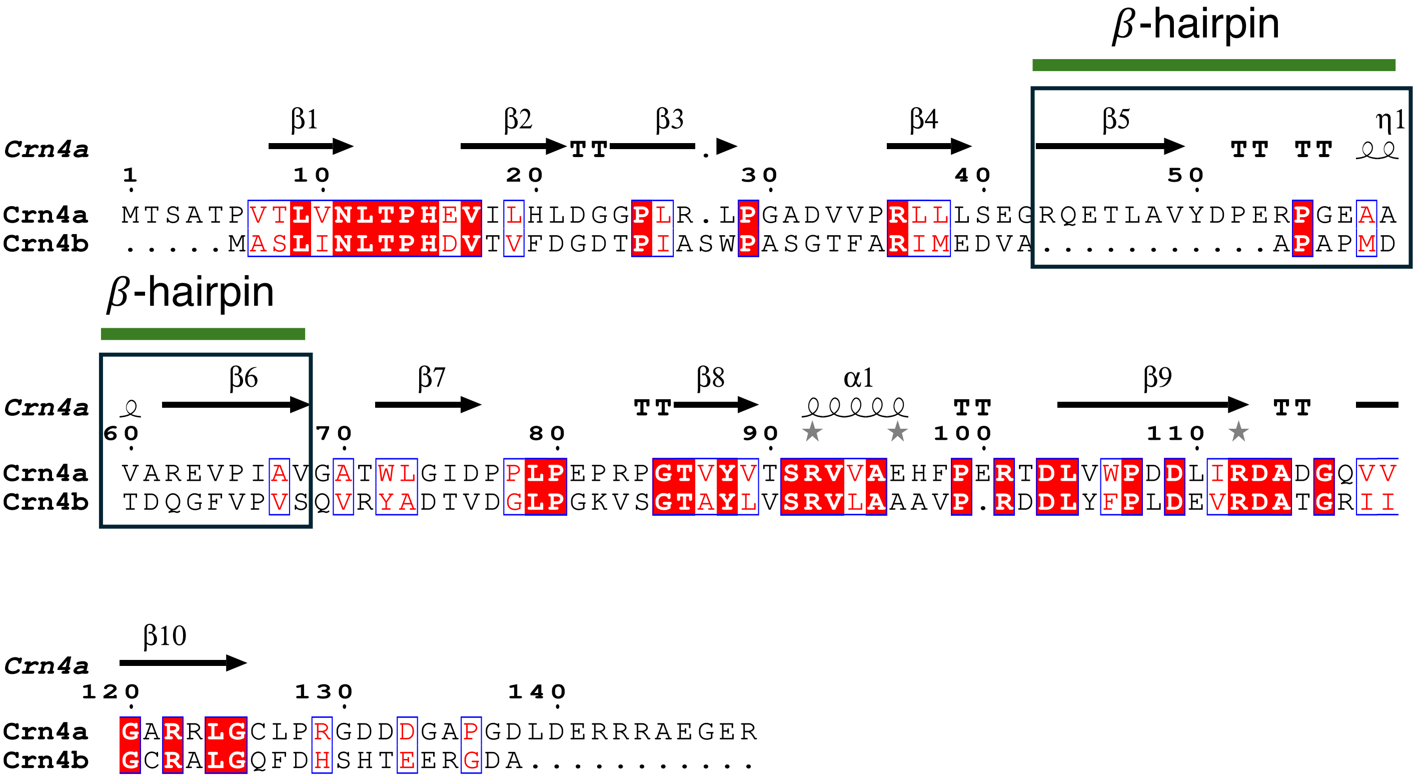

Supplementary Figure 8. Secondary structure-guided sequence alignment of Crn4a and Crn4b.
Secondary structure of Crn4a is displayed above the sequence alignment. The β-hairpin of Crn4 family is highlighted using the black rectangle with green bar on the top, indicating the large insertion in Crn4a. The strict conservation is shown with the white letter on a red background. Figure was generated using ESPript3 (Rober and Gouet, 2014).

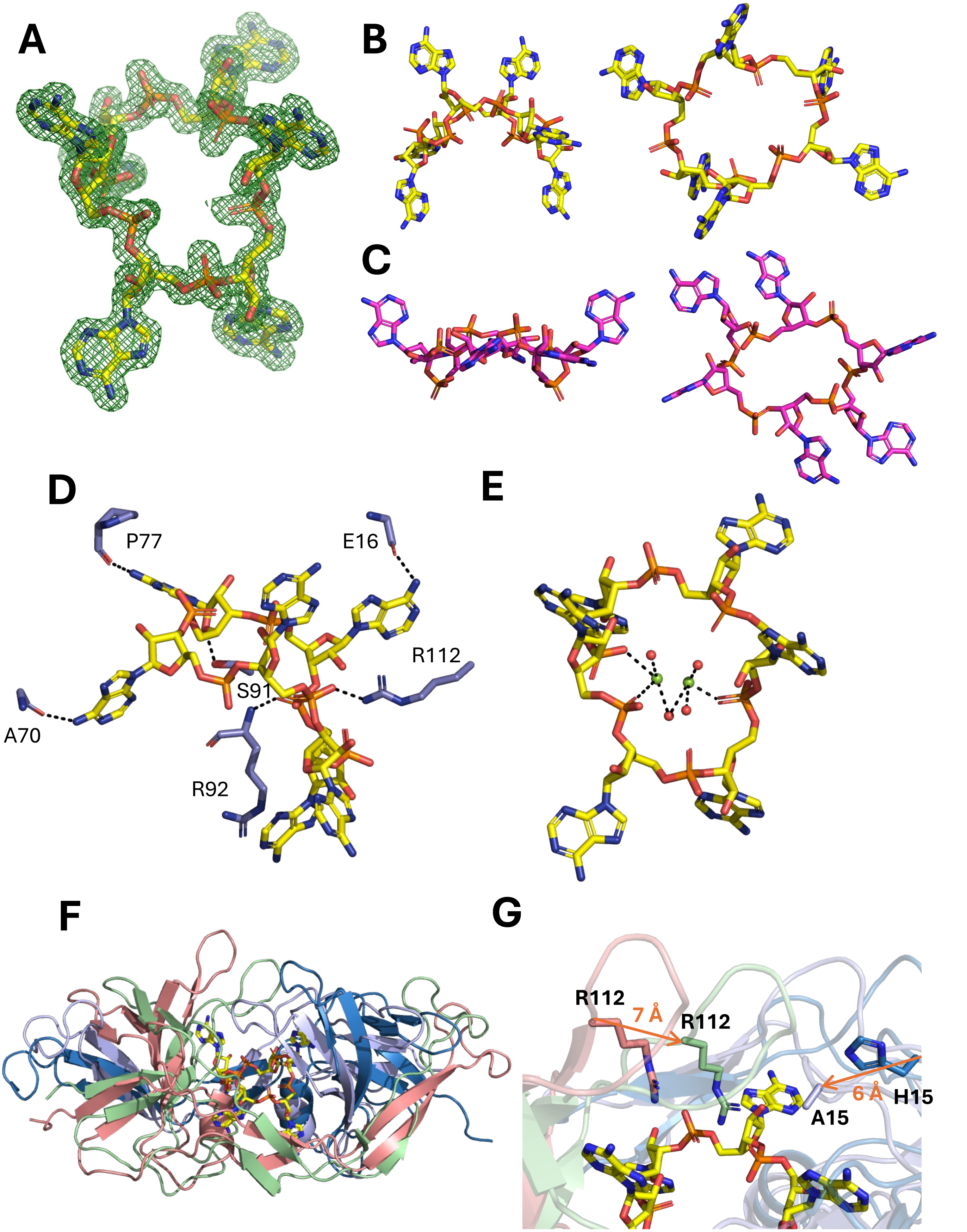
A
B
C
D
P77
E16
R112
S91
A70
R92
E
F
G
R112
7 Å
R112
6 Å
H15
A15

Supplementary Figure 9. Structure and mechanism of Crn4.
A. Structure of cA_6 (yellow sticks) from the complex with Crn4a, with maximum likelihood/σA weighted F_{obs}-F_{calc} electron density map at 3σ shown (green). **B.** Two views of cA_6 (yellow sticks) from the complex with Crn4a. **C.** Two views of cA_6 (magenta sticks) from the complex with the ribonuclease *Streptococcus thermophilus* Csm6. **D.** Active site of Crn4a (mauve) in complex with cA_6 (yellow sticks) highlighting the residues that form hydrogen bonds (black dashed lines), electrostatic (black dashed lines) or pi-pi stacking interactions with cA_6. **E.** Active site of Crn4a showing cA_6 (yellow sticks) forming electrostatic interactions (black dashed lines) with Mg^{2+} ions (green spheres) and their interactions with water molecules (red spheres/black dashed lines). **F.** Superimposition of dimeric apo Crn4a (monomers in pink and blue) and dimeric Crn4a in complex with cA_6 (monomers in mauve and green; cA_6 in yellow sticks). **G.** Closer view of superimposition of dimeric apo Crn4a and dimeric Crn4a in complex with cA_6; colouring as in panel F. H/A15 and R112 are shown on the figure in the colour relevant to the protein molecule on which they are located. The arrows shown in orange indicate the movement of H15 and R112 upon binding of cA_6, by the distance shown.

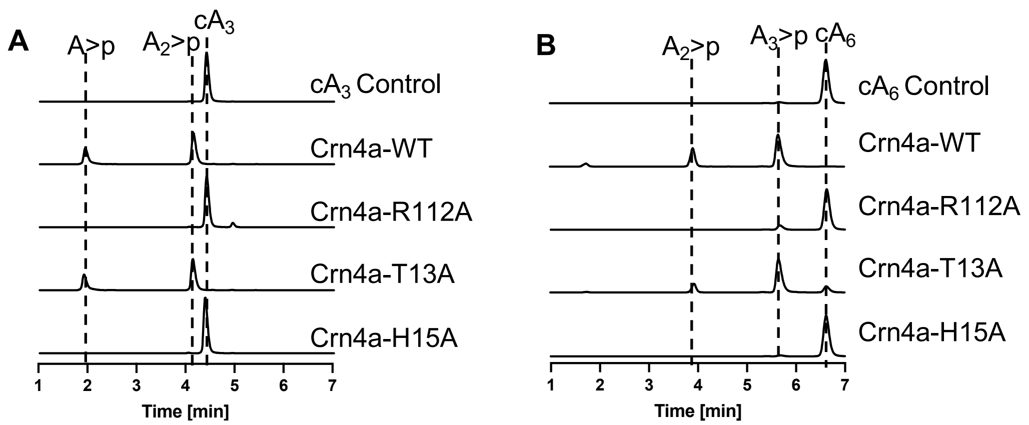

Supplementary Figure 10. Ring nuclease activity of Crn4a WT and variants. HPLC analysis of cA_3 cleavage activity (**A**) and cA_6 cleavage activity (**B**) of Crn4a wild-type and variants. 0.5 μM enzyme is incubated with 100 μM cA_3 or cA_6 at 37 °C for 60 min. Source data are provided as a Source Data file.

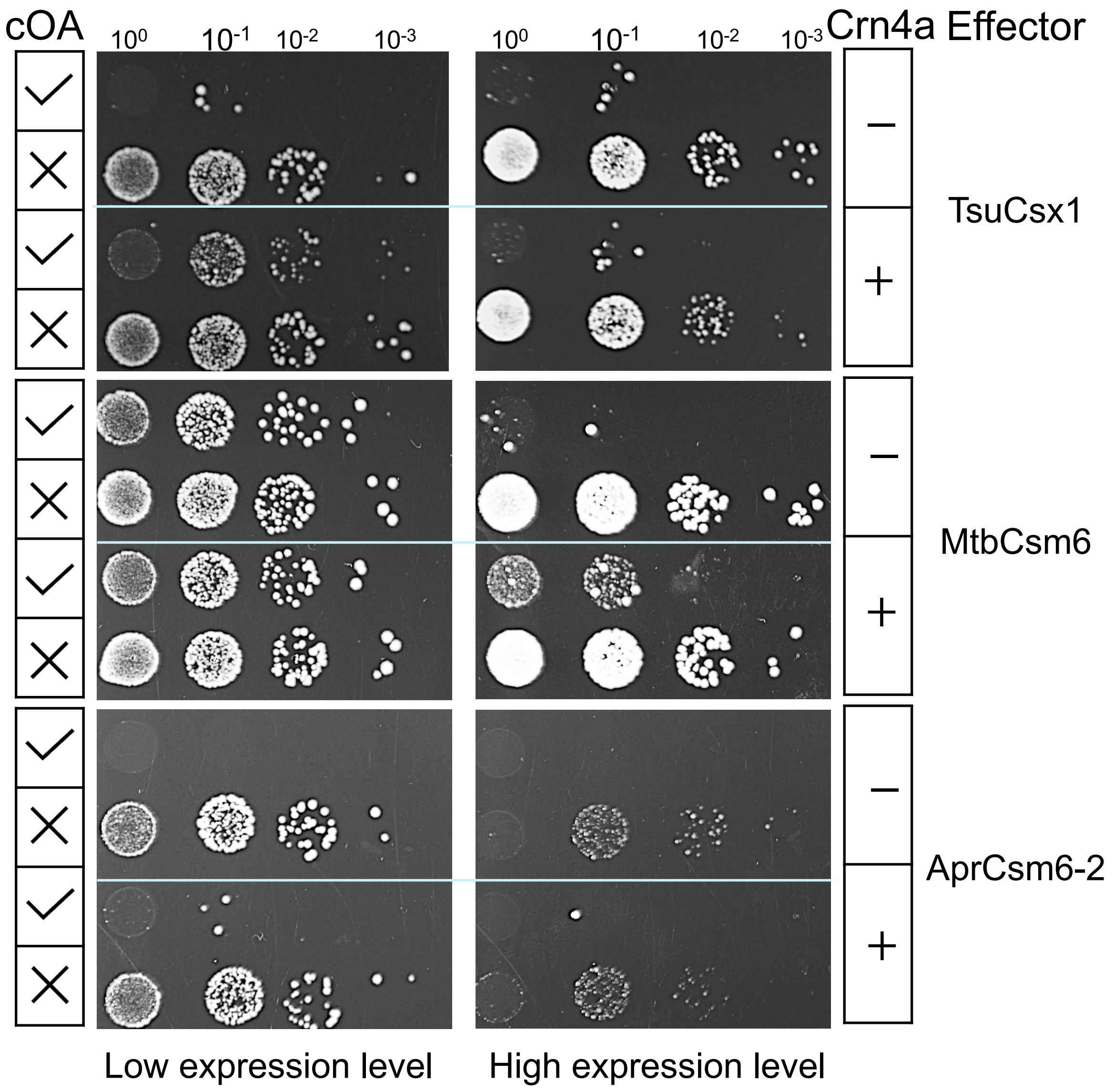

Supplementary Figure 11. Comparison of plasmid immunity in low and high expression levels. C43 cells expressing MtbCsm system programmed with or without cOA production were transformed with plasmids harbouring effectors in the absence or presence of Crn4a. The transformants were selected on LB agar containing 0.2% (w/v) β-lactose and L- arabinose representative as a high expression level, while omitting both inducer presents as a low expression level. The pBAD and T7 promoter used in this systems were always observed a low level of transcription even in the absence of inducer.

Supplementary Table 1. Synthetic genes and mutagenesis primers for Crn4 and Crn4b

Name	Sequence (5’-3’)	Note
**Crn4**	GCGCCCATGGCACATATGACCTCGGCGACACCTGTCACGTT<br>GGTGAACTTAACTCCGCACGAAGTCATCCTTCACCTTGATGG<br>TGGTCCGTTGCGTTTGCCAGGCGCCGATGTCGTGCCTCGCC<br>TTCTGCTTAGTGAGGGACGCCAGGAGACATTAGCGGTGTACG<br>ACCCAGAGCGCCCGGGTGAGGCTGCTGTTGCCCGTGAGGTT<br>CCCATCGCTGTCGGAGCCACCTGGCTTGGCATTGATCCTCCG<br>TTGCCTGAACCCCGTCCCGGTACTGTTTATGTAACATCACGC<br>GTGGTCGCGGAGCACTTCCCTGAACGTACAGATTTGGTCTGG<br>CCCGACGATCTTATTCGTGATGCAGATGGACAGGTAGTGGGC<br>GCCCGTCGTTTGGGGTGCTTGCCGCGCGGTGATGACGACGG<br>AGCCCCTGGTGACTTAGATGAGCGTCGTCGCGCTGAGGGGG<br>AACGC TGACTCGAGGGATCCCGCG	G-block
**Crn4b**	GCGCCCATGGCACATATGGCCTCTTTAATCAACTTAACACCA<br>CACGATGTTACAGTCTTTGATGGGGATACGCCAATCGCGTCC<br>TGGCCAGCCTCTGGCACATTTGCTCGCATCATGGAAGACGTG<br>GCCGCGCCTGCCCCGATGGATACAGATCAGGGCTTCGTACC<br>CGTTTCTCAAGTTCGTTACGCCGACACCGTTGATGGGTTACC<br>CGGCAAGGTGTCGGGGACCGCCTACCTGGTTTCTCGTGTCTT<br>AGCAGCCGCTGTTCCCCGCGACGATCTTTATTTCCCGCTTGA<br>CGAAGTACGCGATGCGACGGGGCGCATCATCGGGTGTCGCG<br>CATTGGGTCAGTTCGACCATAGCCACACTGAGGAACGTGGTG<br>ACGCATGACTCGAGGGATCCCGCG	G-block
**Crn4H15A-fw**	GAACTTAACTCCGGCCGAAGTCATCCTTC	primer
**Crn4H15A-rv**	GAAGGATGACTTCGGCCGGAGTTAAGTTC	primer
**Crn4T13A-fw**	GGTGAACTTAGCTCCGCACGAAGTC	primer
**Crn4T13A-rv**	GACTTCGTGCGGAGCTAAGTTCACC	primer
**Crn4R112A-fw**	GACGATCTTATTGCTGATGCAGATGGAC	primer
**Crn4R112A-rv**	GTCCATCTGCATCAGCAATAAGATCGTC	primer

Supplementary Table 2. Data processing and refinement statistics for the structures of Crn4a, Crn4b, and Crn4a H15A variant in complex with cA_6

	WT Crn4a	Crn4a H15A + cA_6	WT Crn4b
**Data processing**			
**Space group**	P 3_1 2 1	P 2_1	C 1 2 1
**Cell dimensions**			
**a, b, c (Å)**	116.3, 116.3, 38.9	66.3, 102.9, 72.4	46.3, 61.3, 42.7
**α, β, γ (°)**	90, 90, 120	90, 103.9, 90	90, 101.9, 90
**Resolution (Å)**	100.7 – 2.34 (2.38 – 2.34)*	102.9 – 1.44 (1.47 – 1.44)*	30.7 – 1.09 (1.11 – 1.09)*
**R_{merge}**	0.182 (1.229)*	0.068 (2.10)*	0.036 (4.83)*
**I/σ(I)**	10.4 (0.6)*	11.2 (0.3)*	26.0 (0.2)*
**Completeness (%)**	94.9 (69.4)*	100 (99.2)*	79.4 (10.3)*
**Average redundancy**	14.9 (3.7)	6.9 (6.7)*	11.9 (2.6)*
**$CC_{1/2}$**	0.996 (0.375)	0.998 (0.299)*	1.000 (0.228)*
**V_m (Å^3/Da)**	2.37	2.50	2.18
**Solvent (%)**	48.2	50.8	43.7
**Refinement**			
**Unique reflections**	12327 (446)	169690 (8333)	38596 (250)
**R_{work} / R_{free}**	22.2 / 28.2	22.2 / 24.2	16.8 / 18.5
**Geometric deviations**			
**Bonds (Å) / Angles (°)**	0.003 / 1.075	0.004 / 1.164	0.005 / 1.295
**No. atoms (non H)**			
**Protein**	1897	5761	896
**Water**	93	694	98
**cA_6**		396	
**Mg^{2+}**		16	
**B factors (Å^2)**			
**Protein**	43.9	32.1	16.0
**Water**	37.0	36.8	29.9
**cA_6**		20.8	
**Mg^{2+}**		27.1	
**Ramachandran Favoured / outlier (%)**	96.0 / 0	98.4 / 0	99.1 / 0
**Molprobity score / centile (%)**	1.41 / 99	0.93 / 100	1.01 / 98
**Rotamers Favoured / outlier (%)**	94.6 / 0	99.8 / 0.2	97.8 / 0
**PDB Code**	9SMA	9QS9	9R7B

* Values in parentheses are for the high resolution shell

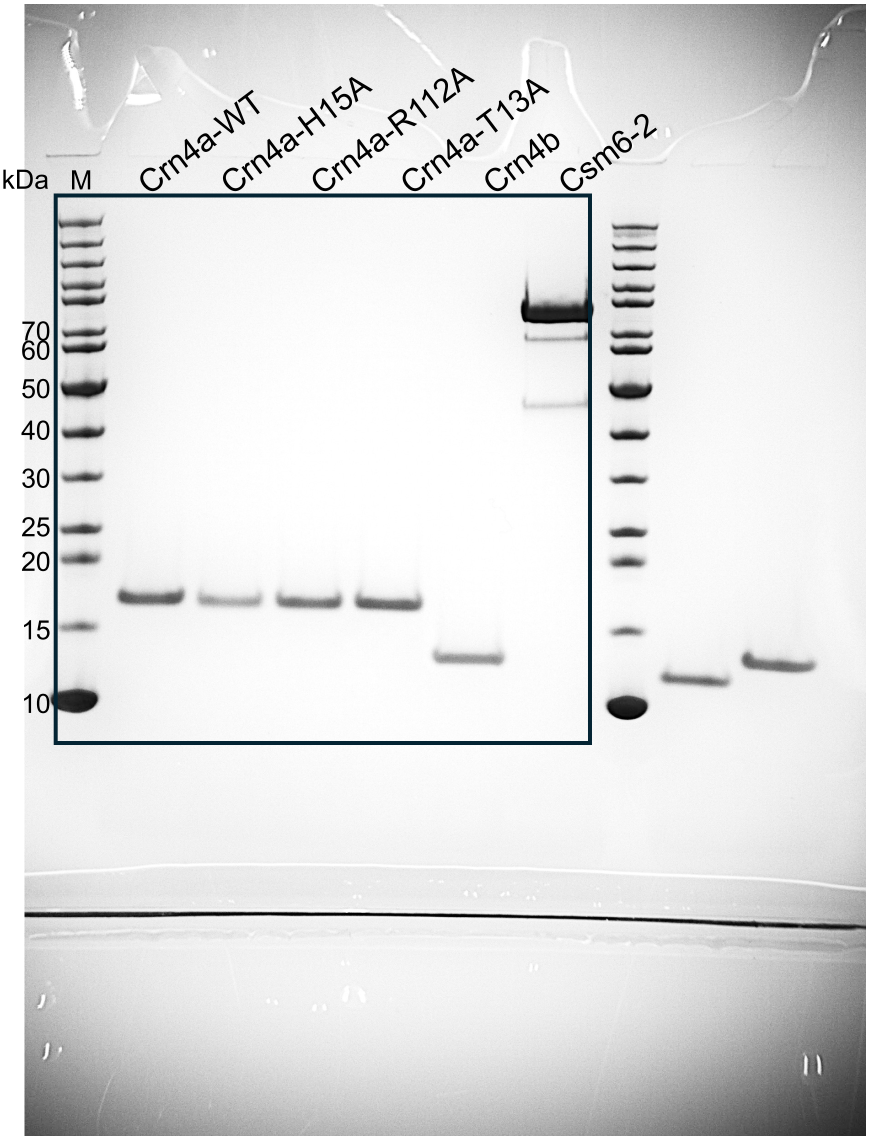

Source Data: uncropped gel for Supplementary Figure 3

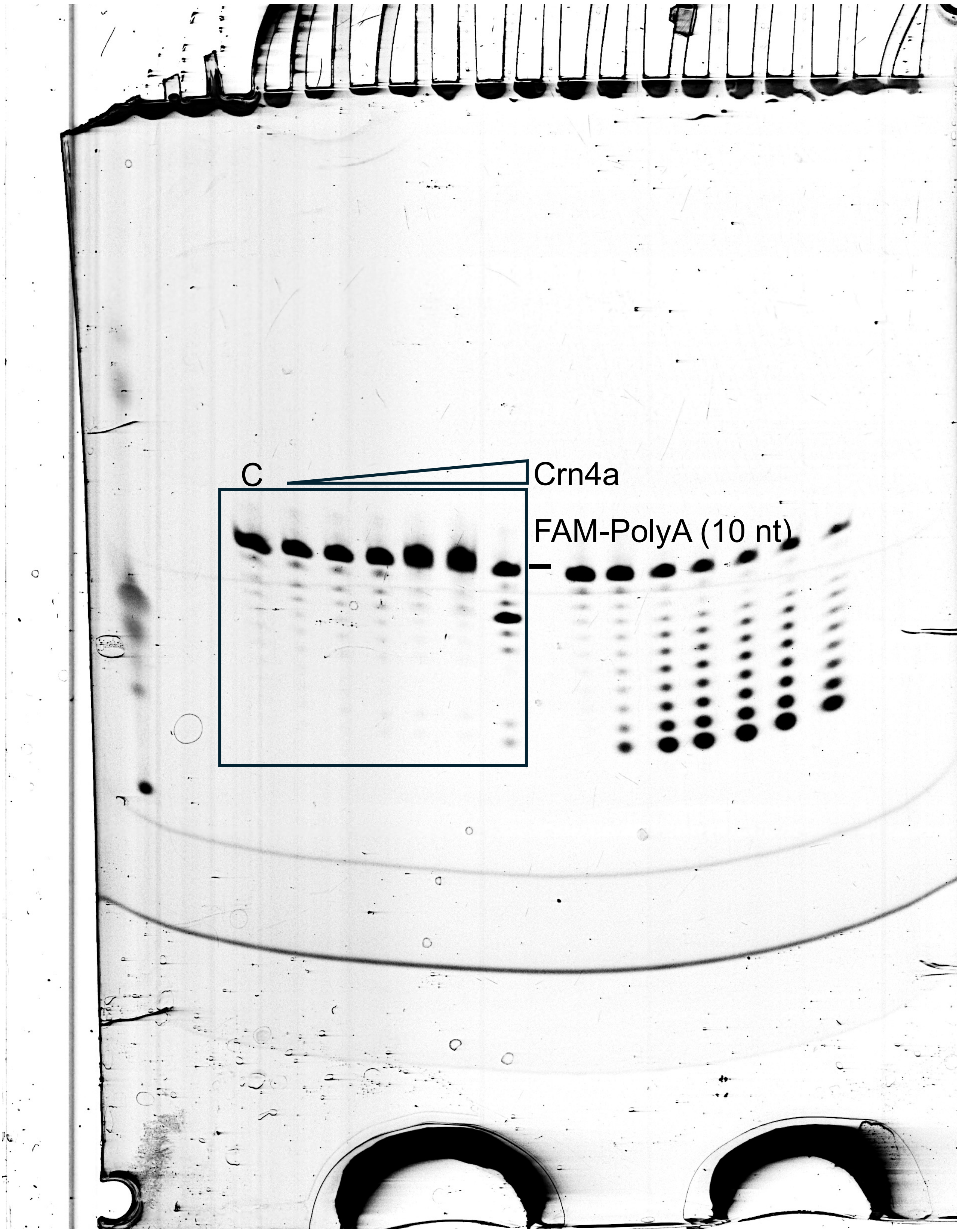

Source Data: uncropped gel for Supplementary Figure 7b
