# Supplementary material for: Structure and mechanism of the broad spectrum CRISPR-associated ring nuclease Crn4
Source: Nat Commun. 2025 Dec 15;17:889. doi: 10.1038/s41467-025-67607-6 (PMC12830947; doi:10.1038/s41467-025-67607-6)
Supplement: Supplementary file 2 — Description of Additional Supplementary Files [file 41467_2025_67607_MOESM2_ESM.pdf]

Description of Additional Supplementary Files

File Name: Supplementary Movie 1
Description: Morph showing the structural changes that accompany cA6 binding by Crn4a, view 1.

File Name: Supplementary Movie 2
Description: Morph showing the structural changes that accompany cA6 binding by Crn4a, view 2.
